# Polyether Block Amide as Host Matrix for Nanocomposite Membranes Applied to Different Sensitive Fields

**DOI:** 10.3390/membranes12111096

**Published:** 2022-11-03

**Authors:** Gabriele Clarizia, Paola Bernardo

**Affiliations:** Institute on Membrane Technology (ITM-CNR), via P. Bucci 17/C, 87036 Rende, CS, Italy

**Keywords:** Pebax, nanocomposites, membranes, pervaporation

## Abstract

The cornerstones of sustainable development require the treatment of wastes or contaminated streams allowing the separation and recycling of useful substances by a more rational use of energy sources. Separation technologies play a prominent role, especially when conducted by inherently environmentally friendly systems such as membrane operations. However, high-performance materials are more and more needed to improve the separative performance of polymeric materials nanocomposites are ideally suited to develop advanced membranes by combining organic polymers with suitable fillers having superior properties. In this area, polyether block amide copolymers (Pebax) are increasingly adopted as host matrices due to their distinctive properties in terms of being lightweight and easy to process, having good resistance to most chemicals, flexibility and high strength. In this light, the present review seeks to provide a comprehensive examination of the progress in the development of Pebax-based nanocomposite films for their application in several sensitive fields, that are challenging and at the same time attractive, including olefin/paraffin separation, pervaporation, water treatment, flexible films for electronics, electromagnetic shielding, antimicrobial surfaces, wound dressing and self-venting packaging. It covers a wide range of materials used as fillers and analyzes the properties of the derived nanocomposites and their performance. The general principles from the choice of the material to the approaches for the heterogeneous phase compatibilization as well as for the performance improvement were also surveyed. From a detailed analysis of the current studies, the most effective strategies to overcome some intrinsic limitations of these nanocomposites are highlighted, providing guidelines for the correlated research.

## 1. Introduction

Nanocomposites are more and more present in our everyday life such as in the automotive sector, construction, anti-corrosion barrier coatings [1] and even in deep space exploration [2]. The market size for nanocomposites, estimated at USD 4.3 billion in 2019, is growing at a compound annual growth rate of 16.3%, thus it is expected to reach USD 14.3 billion in 2027 [3].

Originally, inorganic–organic nanocomposites were developed to take advantage of the lightweight, flexibility and mouldability of a polymer matrix combined with the superior strength, chemical resistance and thermal stability of inorganic additives. However, the mutual interactions between the matrix and the additives result in performance overcoming those described by simple models based on only pure material properties.

Inorganic–organic hybrid membranes, referred to as mixed matrix membranes (MMMs), represent a peculiar category of nanocomposites and are actively investigated for virtually all membrane operations. Indeed, MMMs effectively combine the properties of different materials, allowing to take advantage of even innovative materials (e.g., 2D nanoparticles) in molecular separations [4].

Polyether block amides (PEBAs) are plasticizer-free thermoplastic elastomers (TPEs) [5]. Their macromolecules comprise flexible segments of polyether that are connected to linear and rigid polyamide segments by means of ester groups. The polyamide is nylon 6, nylon 66, nylon 11, nylon 6/11, nylon 12 or nylon 6/12, whereas the polyether block is poly(propylene glycol), poly(tetramethylene ether glycol) or poly(ethylene glycol). Thus, by changing the monomeric block types and ratios, a wide range of physical and mechanical properties can be obtained resulting in customized products. These copolymers were first produced in the early 1980s and are commercially available under the trade name Pebax^®^.

Due to their peculiar chemical and physical properties (lightweight, easy processing, good resistance to most chemicals, flexibility and high strength) [5], Pebax^®^ have been extensively investigated for membrane separation [6,7,8]. The separation of CO_2_ (e.g., from natural gas, from biogas or from flue gases) could be effectively addressed with these membranes that present a good affinity for the CO_2_ molecule due to the polyether block in the copolymer matrix. Therefore, given the interesting properties of the Pebax matrix, Pebax-based nanocomposites are widely studied as membranes to perform the separation of gases and vapours, typically displaying enhanced separation performance [9,10].

However, an increasing number of studies are focusing on films obtained by incorporating nanomaterials in Pebax^®^ copolymers developed for a wide spectrum of applications. According to the reviewed studies, nanomaterials have been synthetized in different shapes and integrated into the polymer matrix, studying their effect on the performance of the nanocomposite films. Approaches to improve the affinity with the hosting polymer matrix include the nanomaterials functionalization or the inclusion of additional components to the formulations. The Pebax^®^ copolymers can be managed both in solution and in solvent-free processes; the resulting films have dense or porous structures. In addition, these block copolymers offer the possibility of selectively confining the additives within a single domain. Therefore, the focus of the present analysis is on the nanocomposites based on Pebax^®^ copolymers, describing their features when applied in challenging areas such as olefin/paraffin separation, pervaporation, fuel cells, antimicrobial coatings, sensing and biomedical applications. 

## 2. Application Domains 

Depending on the composition and PA/PE ratio in the Pebax^®^ copolymers, different properties can be obtained, allowing to address specific applications. The most common Pebax^®^ grades are listed in Table 1, describing their composition.

**Figure 1:**
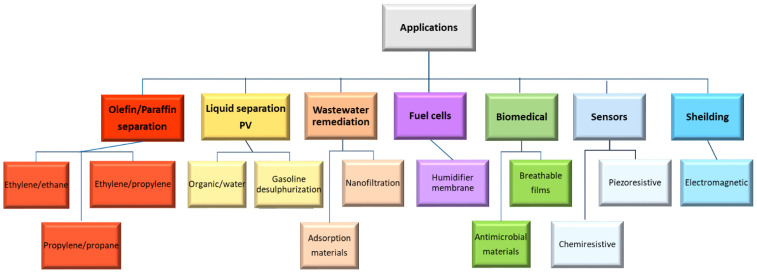
Categorization of Pebax-based nanocomposites on the basis of their application.

Our surveying of the literature was carried out with a particular focus on the last five years, using the Scopus database, and keywords such as Pebax, film and nanocomposite. The nanocomposite MMMs based on Pebax^®^ can be categorized depending on their application domain as described in Figure 1. Recently, the studies on Pebax MMMs for the separation of gas mixtures involving carbon dioxide have been analysed in a separate review that examines the results achieved in the last five years [10]. The present paper examines facilitated transport membranes for olefin/paraffin separation, membranes for liquid separations and fuel cells, biomedical applications, sensors and packaging. Pervaporation (PV) and water filtration represent the principal liquid separations investigated.

## 3. Filler Categories 

Different filler types were exploited for preparing Pebax-based nanocomposites in order to meet the specific requirements of each application. Table 2 summarizes the fillers dispersed into the MMMs for different separations with the adopted loading range, while Table 3 reports the filler types employed for applications not strictly related to separations on the molecular scale.

Typically, the exploited additives are solid nanoparticles that range from inorganic salts (e.g., Ag salts), to metal oxides (TiO_2_, Cu_2_O), carbon-based nanomaterials (graphene, graphene oxide, carbon nanotubes), zeolites, metal–organic frameworks (MOFs), clays and layered double hydroxides (LDHs). The addition of organic molecules and ionic liquids to Pebax matrices was investigated as well. 

Silver salts are the main fillers utilized in olefin/paraffin separation. On the contrary, in PV processes, zeolites, MOFs and carbon-based nanomaterials with different topologies and sizes are widely used. The combination of nanoparticles and ionic liquids was investigated is some studies, as in the olefin/paraffin separation, combining silver salts and ionic liquids. Filler combinations or functionalization are also examined for water treatment and humidification systems. 

The revised studies generally find an optimal value for the filler concentration that is strictly related to the filler type. High amounts of Ag salts are adopted in olefin/paraffin separation, while a low (few percentages) or medium (up to 20 wt%) filler content is optimal for the other separations. In principle, particularly high filler concentrations would result in significant improvements in the transport properties. However, high filler concentrations pose the risk of introducing defects at the interface of heterogeneous phases or losing a portion of the additive in the process. 

## 4. Preparation and Characterization of the Nanocomposites 

The easy processability of Pebax permits to adopt a range of methods for nanocomposite preparation, dissolving the polymer in selected solvents or according to solvent-less procedures. Pebax copolymers can be processed by injection moulding and film extrusion, while melt blending is commonly adopted to produce blends with other polymers. The main techniques in the case of nanocomposite preparation are the following:

- Solution casting,
- Electrospinning,
- Melt electrospinning,
- Hot-melt extrusion,
- Melt compounding,
- Melt blending followed by compression moulding,
- Twin-screw extrusion and compression moulding.

Considering the nanocomposites developed for separation purposes, both supported and self-supported MMMs were investigated, mainly prepared by casting a dope solution (Table 2). 

Regarding the pervaporation process, almost the totality of the nanocomposites is based on the hydrophobic Pebax 2533, while for the olefin/paraffin separation both 1657 and 2533 grades are used. In water treatment and humidification systems also other Pebax grades can be successfully considered. 

In the applications summarized in Table 2, where molecular-based separations are not involved, exclusively self-supported MMMs are investigated with a large variety of filler amount and type. Practically, all Pebax grades are used to incorporate both organic and inorganic fillers.

The nanocomposite membranes are typically characterized using Fourier transform infrared (FT-IR) spectroscopy, scanning electron microscopy (SEM), atomic force microscopy (AFM), X-ray diffraction (XRD), differential scanning calorimetry (DSC), thermogravimetric analysis (TGA) and water contact angle measurements to explore the polymer/filler interactions and to study the effect of the filler incorporation on the morphology, crystalline structure, thermal resistance and wettability of the resulting films. These characterizations clarify the role of the fillers on the performance of the resultant membranes, correlating the membrane properties to the features of the fillers.

## 5. Performance of the Nanocomposites in Different Applications

### 5.1. Olefin/Paraffin Separation

**Figure 2:**
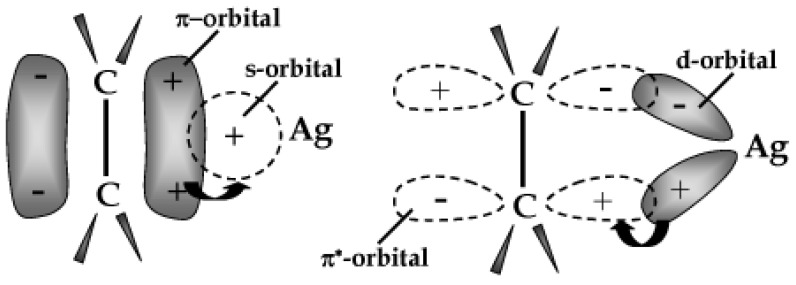
Scheme of the complexation of silver ions and olefins (from ref. [68]).

Several researchers are actively involved in the development of Pebax MMMs for gas separation, particularly focusing on CO_2_ separation from natural gas or from flue gases [10]. However, Pebax-based MMMs were explored to investigate other challenging operations such as the separation of the close boiling point olefins/paraffins. The separation of light olefins from the corresponding paraffins is one of the main separations of industrial interest on a world scale and is typically carried out by the energy and capital-intensive cryogenic distillation process [66]. The global annual production of ethylene and propylene exceeds 200 million tonnes, corresponding to the remarkable quantity of 30 kg per year produced for each inhabitant on the earth [67]. Therefore, the adoption of the membrane separation technology that does not involve a phase change would drastically reduce the energy intensity and the carbon emissions of the process. Membrane modules would decrease both the capital and operating costs of the olefin/paraffin separation [66]. However, neat polymeric membranes are not attractive for these applications, while facilitated transport membranes (e.g., operating on the -complexation) can provide more interesting separation factors. Silver ions are archetypal olefin carriers owing to reversible complexes that they can establish with olefins [68], as schematically shown in Figure 2.

Silver salts (e.g., AgBF_4_) were loaded in Pebax-based membranes as olefin carriers for the challenging olefin/paraffin separation [11]. A ‘salting-out’ behaviour was described for these polymer electrolyte membranes. The addition of a silver salt is expected to enhance the cohesive energy density of the polymer, reducing the affinity for the nonpolar paraffins [69]. However, the low stability over time represents a significant drawback for these membranes. Indeed, due to silver ions reduction, periodic regenerations are required (e.g., by peroxide/acid liquid or vapour phase treatment to oxidise reduced silver carriers within the membrane [11]). Combining a silver salt with an aluminium salt (e.g., Al(NO_3_)_3_) was found to improve the long-term performance of composite membranes due to a stabilising effect on the Ag ions, preventing their reduction [12,13]. Indeed, the interaction between BF_4_^−^ ions and its counter ion, Ag^+^, becomes relatively weaker in the presence of the Al salt and thus the Ag^+^ ions can effectively work as olefin carriers. The use of Pebax as a membrane matrix was envisaged to improve the gas permeability, thanks to the presence of the soft polyether block in the copolymer. However, the permeance was not improved using Pebax^®^2533 (polyamide 20%, polyether 80%) instead of Pebax^®^1657 (polyamide 40%, polyether 60%). A better behaviour in terms of stability was displayed by Pebax^®^1657 compared to 2533. Indeed, in the case of Pebax 2533, FT-IR analysis evidenced as most of the NO_3_^−^ interacts with the polyether segments that constitute the main block in this copolymer [12].

Other approaches to improve the stability of the silver carriers include the addition of an electron acceptor [14] or the use of ionic liquids [15]. The first approach produced Ag nanoparticles (AgNPs) modified using TCNQ (7,7,8,8-tetracyanoquinodimethane) to induce positive charges. The polarized surface of the AgNPs in the Pebax^®^1657 matrix interacted with olefins instead of the silver ions, preventing their reduction, and showing stability for 76 h [14]. However, in the case of the 1-butyl-3-methylimidazolium tetrafluoroborate IL ([BMIM][BF_4_]) added to Pebax^®^5513 (polyamide 60%, polyether 40%), the silver ions were not stabilised since the rigid amide block in the polymer matrix impeded the IL penetration, resulting in a poor propylene/propane separation performance compared to PEO polymers [15].

These studies show that in order to stabilise the metal ions cooperating with the host matrix through Al(NO_3_)_3_, a specific ratio composition of the Pebax polymer matrix is required.

Nanozeolites were dispersed in Pebax^®^2533 for light olefin separation (propylene/ethylene), comparing large pore NaY to small pore NaA zeolites [16]. In agreement with pure gas adsorption, the NaY zeolite was more effective in improving the separation. The optimal separation efficiency was obtained with a concentration of 6 wt% of the NaY particles, achieving a C_3_H_6_/C_2_H_4_ selectivity equal to 13.1 for a mixture containing 80 mol% of propylene.

The selective recovery of hydrofluorocarbons (HFCs) and hydrofluoroolefins (HFO) from exhausted refrigerant mixtures is a particular olefin/paraffin separation. This separation is required to recover and reuse high-global warming potential refrigerant gases, avoiding their emissions. Membranes combining Pebax 1657 with some ionic liquids (e.g., [C_2_mim][SCN], [C_2_mim][BF_4_], [C_2_mim][OTf], [C_2_mim][Tf_2_N]) were prepared with an IL concentration from 20 to 60 wt% [17]. The presence of the ILs, especially at high concentrations, enhanced the mobility of the polymer chains and increased the gas permeability of the smallest molecules R32 and R134a. The permselectivity was affected as well: the largest anions with fluoroalkyl groups (OTf and Tf_2_N) display a lower separation capacity toward the R134a/R1234yf pair, while the small anions (SCN and BF_4_) greatly increase the permselectivity, by more than 100%, with respect to the neat polymer. The authors identified an optimal amount of 40 wt% for [C_2_mim][BF_4_] and [C_2_mim][SCN] to attain the best separation performance and mechanical stability vs. feed pressure, whereas the highest permeability was reached at a higher IL content (60 wt%). 

Table 4 resumes the performance of some Pebax-based MMMs applied to the olefin/paraffin separation in terms of permeance (i.e., pressure normalized flux) and selectivity. 

Ultimately, silver/Pebax-based membranes are capable of enhancing significantly the separation performance in olefin/paraffin separation with respect to the conventional membranes operating according to the solution–diffusion transport mechanism. However, the main drawback of these facilitated transport membranes is the carrier deactivation due to the chemical reduction of silver ions even when incorporated into the polymer bulk. Although some stabilisation strategies were found (e.g., use of H_2_O_2_ [11], or the combination of different salts [12,13]), at the moment, the membrane stability issue is not completely solved.

### 5.2. Pervaporation (PV)

Pervaporation (PV) is recognized as a green operation to carry out the purification process in chemical industries [70,71]. Volatile components in a liquid feed stream are vaporized, transported through a dense membrane and recovered in the permeate stream under vacuum. The species more permeable are those more affine to the membrane material. PV is also capable to separate azeotropic mixtures without using external agents.

The PV application requires dense films. The permeate flux (*J*), thickness-normalized permeate flux (*J_N_*), separation factor (*α*), enrichment factor (*β*) and thickness-normalized pervaporation separation index (PSI) are the main variables employed to evaluate the PV separation performance of the membranes:

(1) $$J=\frac{W}{A\;t}$$

(2) $$J_{N}=l\frac{W}{A\;t}$$

(3) $$\alpha =\frac{\frac{y}{1-y}}{\frac{x}{1-x}}$$

(4) $$\beta =\frac{y}{x}$$

*PSI = J_N_ (α − 1)* (5)

where *W* is the permeate amount collected in the period *t*, *A* is the effective membrane area, *l* is the membrane thickness, *x* and *y* are the weight fraction for the most permeable species in the feed and permeate stream, respectively.

Pebax polymers are available in hydrophilic and hydrophobic grades. Thus, depending on their nature, they can be alternatively applied to dehydration processes, alcohol purification or to the removal of organic contaminants from water. As observed before, the majority of the studies on PV were carried out using the hydrophobic Pebax^®^2533 grade. Liu et al. studied the recovery of ethanol from water by PV using neat Pebax^®^2533 membranes [72]. However, a poor separation performance was reported (separation factor of 2.5) as a consequence of a weak affinity of the polymer with ethanol. Recently, Pebax membranes with enhanced separation performance for the pervaporation of phenol aqueous solution were produced by carefully controlling the solvent evaporation process [73]. In particular, combining a low solvent evaporation rate and high temperature assisted the formation of a uniform membrane with an augmented number of amorphous domains. 

Improved performance can be achieved by incorporating appropriate fillers in the polymer matrix. Therefore, the combination of suited nanofillers with Pebax materials in MMMs was exploited in order to improve their performance in PV. Both non-porous and porous nanoparticles were considered and the following paragraphs will present the role of the different fillers. 

The revised papers analyse the microstructure-performance relationships of MMMs. The effect of the operating temperature will be discussed as well. Typically, an increase in the feed temperature results in enhanced segmental mobility of the polymer macromolecules and free volume of the membrane, facilitating mass transport. Accordingly, permeation flux increases, while the separation factor of the MMMs is improved when the small molecules in the feed stream move quicker, diffusing in the membrane at a higher velocity. 

However, in the case of MMMs applied to PV, there are several issues that can negatively affect the separation performance of the membranes. They include the poor adhesion between the phases with the formation of interfacial voids that are non-selective, the occurrence of particle clusters, the filler segregation due to their sedimentation, the polymer chains rigidification and the pore blockage for porous fillers. 

#### 5.2.1. PV MMMs Comprising Non-Porous Fillers 

Studies on nanocomposites comprising inorganic fillers, such as titania (TiO_2_) [74] or silanized alumina (Al_2_O_3_) nanoparticles [75], demonstrated enhanced thermal and mechanical stability. The nanocomposites including up to 60 wt% of Titanium microparticles in Pebax 5533, obtained by twin-screw extrusion and compression moulding, showed enhanced mechanical properties as well as surface roughness resulting in lower surface energy [74]. The silanized Al_2_O_3_ nanoparticles improved the thermo-oxidative stability of the copolymer [27]. The thermal analysis coupled to the FT-IR indicates that the soft PTMO segment was constrained by the fillers, as evidenced by the increase in *T*_g_ of this component [75]. Instead, the crystallinity for the hard PA12 domains was reduced owing to the chemical interaction with the functional groups on the alumina surface. Titania represents the main non-porous filler dispersed in Pebax to prepare PV MMMs. Indeed, the presence of hydroxyl groups on the TiO_2_ surface improves the membrane wettability. 

Thin-film nanocomposite (TFN) membranes were developed dispersing TiO_2_ nanoparticles in a Pebax layer that is deposited onto a polymeric porous support. PDMS supports coated with Pebax loaded with TiO_2_ nanoparticles (up to 1.5 wt%) were applied to the acetone/water separation [18]. This separation is involved in the treatment of wastewater generated by different industrial units (e.g., dyeing, ink production, glazing). The optimal performance was obtained at a titania loading of 0.75% that resulted in well-dispersed nanoparticles, while at larger concentrations the formation of aggregates was observed.

In another study, TiO_2_ was deposited by dip-coating on a composite membrane comprising a porous PES/PSf blend support with a top layer of Pebax and the resulting membrane was applied to the PV removal of humic acid from water [19]. The titania particles strongly adhered to the membrane surface thanks to the hydrogen bonding between their hydroxyl groups and the amide groups in Pebax and the coating was stable even after the membrane washing. Both the hydrophilicity and roughness of the membranes were enhanced by the titania coating that contributed to improving the antifouling properties. The optimum concentration of nanoparticles was 0.03 wt% for the flux enhancement and 0.01 wt% for the humic acid rejection. At higher TiO_2_ loading (0.05 wt%), microscopy images evidenced nanoparticle agglomeration that hindered the contact with the active adsorption sites. A more concentrated feed solution results in a lower flux due to concentration polarization phenomena. On the other hand, a high feed pressure increases the water flux as a consequence of a higher driving force, but also decreases the humic acid rejection because of an accumulation of acid molecules on the membrane surface, forced to pass through it. 

#### 5.2.2. PV MMMs Comprising Porous Fillers 

##### Zeolites

Zeolites, microporous aluminium silicates, are characterized by well-defined frameworks with openings that are close to the molecular dimensions of permanent gases and some vapours [76]. 

Zeolites with different topologies and, thus, different pore sizes and framework architectures, were incorporated into Pebax for preparing PV membranes [20,21,22,23]. 

Large pore NaX nanozeolites were homogeneously dispersed at 2 wt% into the hydrophobic Pebax^®^2533 [20]. The separation efficiency of toluene from aqueous solutions was improved, by enhancing the permeation flux, especially at high toluene concentrations. The degree of swelling (DS) of the membranes increases with the toluene concentration in the feed; however, thicker membranes (from 25 μm to 75 μm) are more resistant to the swelling [20].

Medium pore ZSM-5 zeolites, having a large size (3∼5 μm in size), were dispersed in Pebax obtaining self-supported MMMs [22]. Instead, ZSM-5 nanoparticles were used to prepare dual layer membranes having a nanocomposite Pebax active layer supported by a polyether sulfone (PES) membrane [23]. Both membrane types were applied to the ethyl acetate/water PV separation. The ZSM-5 zeolites enhanced the hydrophobicity of the Pebax-based MMMs. The best separation performance was obtained at a loading of 10 wt% ZSM-5 in both cases. However, better results were achieved by reducing the particle size and the membrane thickness [23]. Both feed concentration and temperature positively affect the total permeation flux and separation factor. Fluid dynamic conditions significantly influence the separation: turbulent conditions result in improved performance with respect to laminar flow [23]. The same authors produced composite membranes with enhanced performance by using an ionic liquid/Pebax intermediate layer coated on a porous support and protected by a Pebax/ZSM-5 top layer to avoid the IL ([Hmim][PF_6_]) leaching [24]. The PSI of the triple-layer membranes was 1.5 times that of the neat PEBA membrane.

Monocrystalline silicalite-1 nanoparticles (having the same topology of ZSM5 zeolite, but no aluminium in the structure) with a hollow structure (HMS) were uniformly dispersed in a Pebax matrix and tested for the gasoline desulfurization via PV [21]. The fillers interrupted the crystalline regions in the polymer matrix and increased the membrane free volume. In addition, their hierarchical structure simultaneously enhances the flux and the selectivity: the inner pores of the zeolites favoured the molecular diffusion, increasing the permeate flux; on the other hand, the micropores on the HMS shell improve the selectivity due to a sieving effect. Smaller particles (200 nm) were more beneficial than larger particles (350 nm) and the related MMMs surpassed the upper bound of the state-of-the-art reported for polymeric membranes. Fillers with smaller sizes have larger surface areas and provide more free-volume spaces at the interface with the polymer macromolecules. Further advantages were achieved as the improvement of swelling resistance, thermal and long-term operation stability.

##### Metal–Organic Frameworks (MOFs)

Metal–organic frameworks (MOFs) are crystalline solids in which metal ions or clusters are linked through molecular bridges [77]. They combine a straightforward synthesis with tailorable pore size and chemical functionality, resulting in particularly attractive molecular separation in membranes. MOFs were incorporated in Pebax membranes developed for PV separation. 

Flat sheet Pebax 2533 MMMs including superhydrophobic and organophilic zeolitic imidazolate frameworks (ZIF-71) particles were successfully applied to recover biobutanol from acetone–butanol–ethanol (ABE) broth of a batch anaerobic fermentation [25]. The priming approach was used by Liu et al. to prevent particles from agglomeration and interfacial voids [25]. The selective permeation of the membrane, following the order n-butanol > acetone > ethanol, is primarily due to a preferential sorption mechanism. The MMMs are more hydrophobic than the unfilled membranes, showing a high affinity for n-butanol, but not so much that they are easily fouled in the fermentation broth. The separation factor and selectivity in the mixture of the MMMs increased with the ZIF loading. With the increase of ZIF-71 loading, glass transition temperature (*T_g_*) firstly decreases and then increases. The addition of a small amount of ZIF-71 particles disrupts the inherent organization of the polymer chains and enhances the accessible free volume in the matrix. The highest permeability, in a binary n-butanol–water mixture, was achieved at 20 wt% of filler as chain packing of the MMMs is looser than the pure PEBA membrane and butanol molecules can diffuse freely through the pores of the ZIF-71 cages. When the loading reached 25 wt%, the partial polymer chain rigidification, combined with the tortuous pathways across the membrane due to the large filler loading, reduces the permeation flux. Membranes were stable for 100 h of continuous PV experiment in ABE fermentation broth with separation factor and flux similar to those obtained using a model ABE solution in which inactive microbial cells and several other metabolic compounds are absent. Interestingly, the MMMs, even if more hydrophobic than the original PEBA, are not fouled in the real fermentation conditions [25]. 

MMMs without interphase defects were prepared by encapsulating Zn(BDC)(TED)_0.5_ (BDC = benzenedicarboxylate, TED = triethylenediamine) particles up to 20 wt% into Pebax and tested for bio-butanol recovery via PV [26]. The good contact at the polymer/filler interface was obtained via a priming procedure, as confirmed by the enhanced mechanical properties coupled with decreased surface free energy. 

Another study, proposed the modification of large pore MOFs, the MIL-101, via covalent grafting of designed hydrophobic ionic liquids (ILs) [28]. The ILs-modified MIL-101 was dispersed in Pebax, preparing MMMs for the ethyl acetate pervaporation. The MOF pore structure was successfully tuned through the ILs incorporation within the large cages and over the surface of MIL-101 particles, with a pore size transition from mesoporous to microporous. In addition, the modified surface properties of MIL-101 inhibited the formation of larger aggregates with no noticeable defects in the MMMs.

Hollow fibre mixed matrix composite membranes were prepared by a facile dip-coating method using ceramic supports and applied for recovering ethanol from an aqueous solution via pervaporation [27]. The MMM layer comprises RHO-[Zn(eim)_2_] (MAF-6, Heim = 2-ethylimidazole) nanoparticles that remarkably enhanced both flux and separation factor, with respect to the pure PEBA-based membranes.

##### Other Porous Fillers 

Li et al. modified MCM-41, a mesoporous silica molecular sieve, using two ILs [29]. The ILs, 1-ethyl-3-vinylimidazolium bis[(trifluoromethyl)sulfonyl]imide ([EVIM][Tf_2_N], IL1) and N-octyl-pyridinium bis[(trifluoromethyl)sulfonyl]imide ([OMPY][Tf_2_N], IL2), are both hydrophobic due to the presence of two trifluoromethyl (-CF_3_) groups in the anion. The modified fillers were loaded within a Pebax matrix, preparing membranes for butanol/water PV. The mesoporous structure of the molecular sieves was preserved after the IL modification, as shown by XRD, TEM and BET analyses, thus it facilitated butanol and water diffusion in the membrane, significantly improving the permeation flux. The IL-modified particles enhanced the membranes’ hydrophobicity, particularly in the case of the MOFs modified with IL2 which present a longer alkyl chain on its cation. Accordingly, the presence of the IL-modified MCM-41 promoted the preferential sorption of butanol, enhancing also the separation factor of the MMMs. The optimum loading was 5 wt% for both fillers, while their agglomeration was observed at 10 wt%. The MCM-41-IL2 provided membranes with slightly better performance than MCM-41-IL1, with a higher degree of swelling and affinity for butanol, showing also high stability within 100 h of operation. The PV performance was improved at a high feed temperature, due to the enhanced diffusion for the small species and membrane free volume [29].

The polyhedral oligomeric silsesquioxanes (POSS), having an inorganic silica-like core surrounded by organic groups, were employed for preparing PV membranes to recover ethanol from water [30]. The studied POSSs, octa(3-hydroxy-3-methylbutyldimethylsiloxy) (AL0136) and disilanolisobutyl (SO1440), increased both permeation flux and separation factor with respect to the neat polymer. The optimum loading was 2 wt% for both fillers. However, AL0136, with a higher affinity towards ethanol, gave better performance than SO1440. A typical trend of a higher flux combined with poor selectivity was observed as ethanol feed concentration increased. A favourable influence of operating temperature on the separation factor was reported.

A highly stable porous silica (Santa Barbara Amorphous-15, SBA-15) was exploited for PV gasoline desulfurization [78]. This hierarchical porous filler combines mesopores and micropores. The mesopores in SBA-15 operate as highly permeable pathways for molecular transport, while the micropores on the wall of SBA-15 provide molecular sieving that improves the selectivity. The optimal SBA-15 content was 6% since at larger concentrations severe particle agglomeration resulted in tortuous pathways for molecular transport.

Cu_2_O nanocrystals were incorporated into PEBA for recovering pyridine from an aqueous solution since the lone pair of electrons of the pyridine nitrogen atom can form a complex with some metal ions such as Cu^+^ or Ag^+^ [31]. The hydrophobic nanoparticles increased the membrane affinity with the organic permeant which is bulkier than the water molecule. The optimal loading was 6%, while at larger loadings of Cu_2_O the particle agglomeration produced non-selective voids, negatively influencing the degree of swelling. The total flux decreased with increasing Cu_2_O loading, while the separation factor initially rises and then decreases. The feed temperature positively affects the separation.

#### 5.2.3. Two-Dimensional Nanosheets 

Two-dimensional (2D) materials have emerged as a promising materials platform, endowing MMMs with facilitated transport pathways that result in a highly selective passage of small molecules, thus effectively discriminating species with similar physical properties [79]. Studies on nanocomposites comprising 2D nanosheets for gas separation evidenced as the filler aspect ratio influences the gas permeability through the MMMs, not only the volume fraction [80]. Indeed, 2D fillers can increase membrane selectivity due to the strongly distorted diffusion paths within the polymer matrix, particularly when the 2D nanosheets align parallel to the membrane surface. 

**Figure 3:**
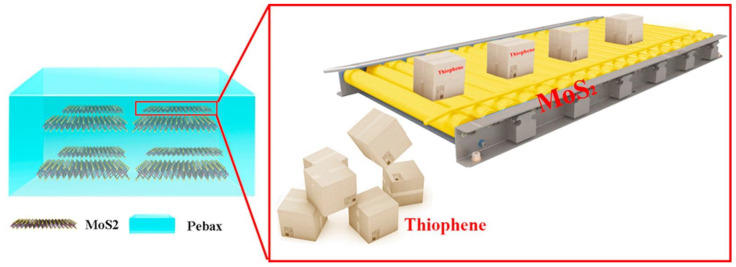
Scheme of an MMM comprising MoS_2_ 2D nanosheets that create a continuous facilitated transport pathway for thiophene (from ref. [32]).

Molybdenum disulfide (MoS_2_) nanosheets were dispersed within a Pebax matrix, producing MMMs for gasoline desulfurization [32]. The Pebax/MoS_2_ MMM layer was deposited on a PSf ultrafiltration membrane by spin-coating. The large and clean basal plane of the MoS_2_ nanosheets provides continuous facilitated transport pathways (Figure 3). Studying the removal of thiophene from model n-octane-thiophene mixtures, the high diffusion coefficient for thiophene molecules was measured and correlated to the moderate binding energy of thiophene on the basal plane of MoS_2_.

The high hydrophobicity of MoS_2_ was exploited in another study in which the nanosheets were loaded up to 20 wt% into the hydrophobic Pebax 2533 and applied to the PV separation of pyridine from water [33]. The total permeation flux of the membrane decreases with the increase of MoS_2_ loading, while the separation factor increases first and then decreases, with a maximum of 10 wt%. Indeed, a larger MoS_2_ loading leads to a rigidification of the polymer matrix, improving the thermal stability and depressing the permeation flux. On the other hand, the hydrophobic particles reduced the water flux, promoting the diffusion of the larger pyridine molecules. Accordingly, the swelling degree of the membranes is reduced, favourably influencing the separation factor. Agglomeration of MoS_2_ particles was found at loadings > 10 wt%, creating interface gaps between the filler and the polymer that are non-selective. The feed temperature positively affects both permeate flux and separation factor, while a higher feed concentration improves only the permeation flux. At high temperatures, the free volume of the hybrid membrane increases, resulting in an easier transport of large-sized pyridine molecules. Instead, at high pyridine concentration, the swelling degree of the membrane increases and the small water molecules are easily transported.

Graphene, constituted by carbon atoms arranged in hexagonal frameworks that compose monolayers [81], was extensively used in MMMs allowing noticeable improvement of the membrane performance even at low concentrations (i.e., <1 wt% [82]). The hydrophobic graphene particles were considered to increase the membrane hydrophobicity, producing Pebax/graphene membranes with remarkably better performance in the PV separation of isopropanol (IPA) from water compared to neat Pebax membranes [34,83,84]. 

The addition of graphene to the hydrophobic Pebax 2533 up to 2 wt%, increased thermal stability, tensile strength and crystallinity [83]. The enhanced hydrophobicity leads to hindered water diffusion, improving the separation factor. The strong polymer/filler interaction limited the polymer mobility and reduced the swelling degree for the membrane, while the IPA adsorption raised according to the hydrophobic nature of the graphene. The best PV results were obtained at a graphene content of 1.5 wt%, with fully exfoliated and well-dispersed graphene nanoplatelets as shown by XRD. At higher filler concentrations, the membranes were less mechanical resistant due to agglomeration phenomena that reduced flux, swelling degree and separation factor [34].

**Figure 4:**
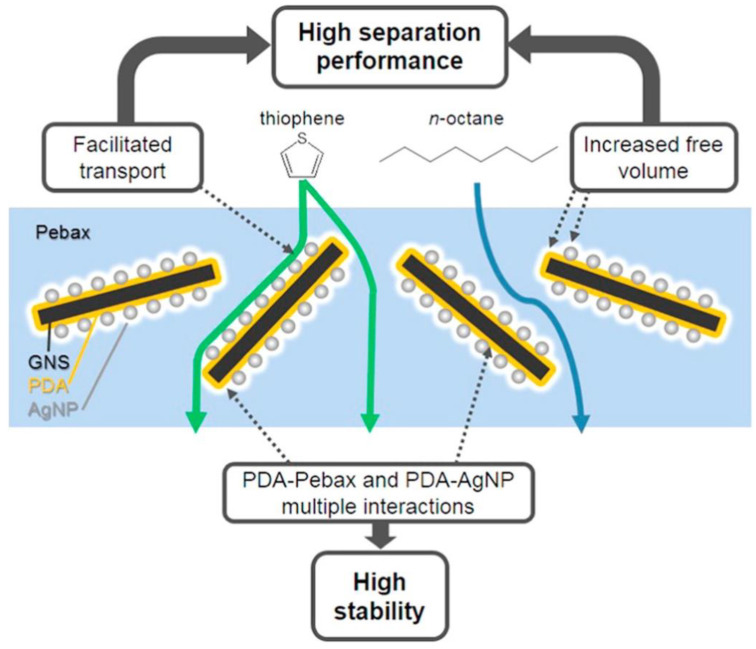
Scheme of an MMM comprising graphene nanosheets (GNS) functionalized by polydopamine (PDA) coating and then loaded with silver nanoparticles (AgNP) (from ref. [35]).

Multifunctional fillers based on graphene were incorporated into Pebax 2533 to study gasoline pervaporative desulfurization [35]. Graphene nanosheets (GNS) were coated with polydopamine (PDA) and then loaded with silver nanoparticles (AgNP) before their dispersion in Pebax. The use of PDA increased the hydrophobicity of graphene, improves the interfacial compatibility between GNS and Pebax and has an adhesive effect on AgNPs. This arrangement favourably combines separation performance and swelling resistance, key factors to separate organic-organic mixtures, taking advantage of the exclusive properties of each component of the composite membrane. The concurrent different transport mechanisms (solution–diffusion and facilitated transport, Figure 4) enhance the separation performance. Indeed, the fillers decrease the crystallinity and lead to an increased FFV without stacking the crystalline polymer chains and adhesion among the heterogeneous components. The graphene nanosheets increase the membrane FFV since they interfere with the chain stacking of crystalline PA segments. The AgNPs are capable to increase the d-spacing of graphene, meanwhile, they provide facilitated transport towards thiophene. On the other side, the complex nature of the Pebax copolymer combines a high permselectivity towards the thiophene due to the flexible PE segments and high anti-swelling stability against model gasoline due to the rigid PA segments. The best performance in terms of permeation flux and enrichment factor is attained at a loading of 6 wt%, while a five-fold increase in flux is measured as the temperature rises from 40 to 70 °C with a small drop in the enrichment. 

Graphene oxide (GO) is usually synthesised by chemical oxidation and exfoliation of graphite, (e.g., via the Hummers method) [85]. Contrary to graphene, GO is highly hydrophilic since the oxidation generates several oxygen-containing groups (hydroxyl, epoxide and carboxylic groups) on the GO surface. Among the revised papers, the only hydrophilic separation, namely IPA dehydration, was studied using PV membranes produced by inserting GO in both the selective hydrophilic layer and the porous hydrophobic support [36]. GO, embedded in the Pebax 1657 selective hydrophilic layer and in the PSf substrate, creates a mutual bridge between the two membrane layers. Accordingly, water molecules, selectively absorbed at the dense selective surface, can diffuse through to the porous support via GO polar groups. A permeate stream without IPA traces was achieved using only 0.4 wt% of GO in the membranes.

Recent studies focused on the synthetic approaches capable of increasing the aspect ratio of MOF nanoparticles, leading to 2D nanosheets [37,38]. These works showed how to control the morphology and preferential orientation of 2D MOFs and could contribute to the design of high-performance membranes for various applications. 

Morphology-tailored zeolitic imidazolate frameworks (ZIFs) were precisely synthesised and dispersed into Pebax obtaining MMMs for the PV separation of phenol/water mixtures [37]. By increasing the precursor concentrations, more bridged N–H∙∙∙N hydrogen bonds generated the ZIF-L nanosheets with a leaf-like shape [37]. Thin-film composite membranes were obtained by spin-coating and spreading a Pebax/ZIF-L solution on PVDF porous supports. The synergistic effect of centrifugal and gravitational forces resulted in the horizontal orientation of the leaf-like nanosheets to the membrane surface, as assessed by XRD at loadings below 4 wt%. Highly permeable MMMs were obtained. Moreover, the high aspect ratio ZLNs promoted phenol transport, limiting that of water. The fillers reduced the surface free energy of Pebax, causing a higher antifouling capacity and ensuring long-term stability in realistic conditions. 

**Figure 5:**
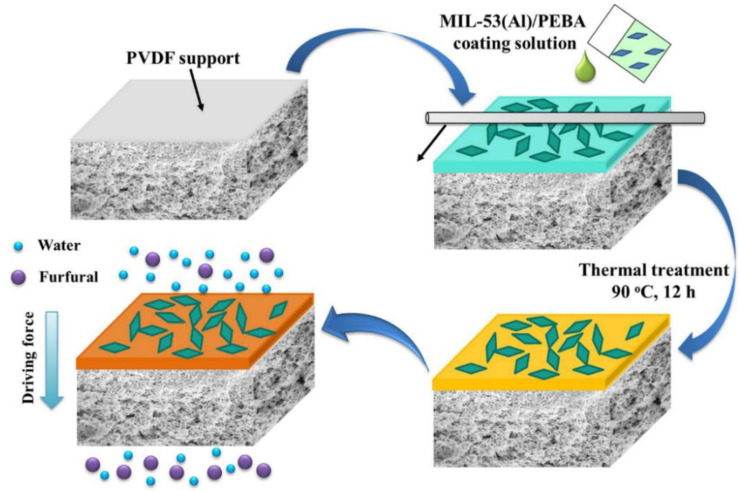
MMMs incorporating MIL-53(Al) MOFs, synthesised by microwave with a coin-like morphology, for furfural/water separation (from ref. [38]).

MIL-53(Al) MOFs, synthesised by microwave with a coin-like morphology, were loaded in Pebax 2533 for the separation of furfural from aqueous solutions (Figure 5) [38]. 

The microwave synthesis remarkably reduced the synthesis times required to produce the MOFs, passing from several days to minutes. Nevertheless, the described preparation method allows for controlling the particle size and morphology. Good interfacial compatibility was observed owing to the chelating effect between the aluminium nodes of MOFs and the ether groups of PEBA, resulting in enhanced thermal stability. The MOFs, being hydrophobic and porous, boosted the affinity for furfural and the PV performances of the MMMs. The optimal performance was obtained using the coin-like particles (referred to as MAPs—16.7, 0.57 μm in size) due to homogeneous distribution and molecular sieving provided by MIL-53(Al). A high PV performance was evidenced at a loading of 15% (total flux of 3800 g∙m^−2^∙h^−1^ and a satisfactory furfural/water separation factor of 50.2). The MMMs’ performances were stable during the long-term operation (200 h) in a simulated hydrolysate containing furfural/acetic acid/water.

The 2D ultrathin metal–organic framework nanosheets (Co-UMOFNs) were loaded into Pebax coating PVDF supports to prepare composite MMMs for the PV removal of phenol from aqueous solutions [39]. The water flux was reduced and the phenol separation factor increased. Indeed, the fillers enhanced the membrane hydrophobicity. As observed in the MMMs loaded with other 2D fillers (e.g., MoS_2_ [33] or graphene [34]), the crystallinity of the MMMs was increased, particularly that for the PE block. In addition, the filler morphology resulted in a “brick and mortar” architecture, resulting in larger transport resistance for water due to the increased tortuosity [39]. 

#### 5.2.4. Organic Fillers

Organic fillers were considered for producing MMMs in order to obtain a good interfacial affinity between fillers and polymer.

Dense membranes for the ethanol separation from water were prepared by incorporating organic fillers (i.e., 4-(trifluoromethyl)-N-(pyridine-2-yl)benzamide (denoted as F1) and 4-(dimethylamino)-N-(pyridine-2-yl)benzamide (denoted as F2)) [40]. These fillers enhanced the hydrophobicity of the membrane surface compared to the neat polymer, resulting in surfaces with a high dispersive free-energy component and roughness. The optimal filler content was 2.5 wt% of F1 and 10 wt% of F2; however, the membranes containing 2.5 wt% of F1 gave the best PV performance, also compared to other PEBA MMMs. 

Facilitated transport membranes were prepared by loading Pebax with Covalent Organic Frameworks (COFs) that were used as carriers to immobilize metal ions [41]. PSf supports were coated by a Pebax MMM layer and used for the gasoline desulfurization by PV. The filler, Ag^+^@SNW-1, is a COF with amino groups and a high amount of silver ions (48 wt%). Good interfacial compatibility between filler and polymer was obtained due to the organic nature of SNW-1. The channels within the SNW-1 filler introduced extra free-volume cavities within the membrane, improving the diffusion rate of the penetrants. On the other hand, the facilitated transport sites in the membranes resulted in enhanced sorption selectivity due to the reversible π-complexation interaction between Ag^+^ and thiophene molecules [41]. Therefore, the hybrid membranes exhibited simultaneously enhanced permeation flux and selectivity. In addition, the MMMs displayed better anti-swelling behaviour, resulting in extended long-term stability (144 h). At higher feed temperature and thiophene concentration, the permeation was improved flux, while the MMMs enrichment factor was lowered.

Table 5, Table 6 and Table 7 summarize the performance of different MMM types in pervaporation processes applied to the most urgent organic-liquid separations. The considered separations belong to the hydrophobic PV which is the selective removal of organic molecules from aqueous solutions. However, the most common pervaporation process is dehydration using hydrophilic membranes [86]. 

Each table collects the data obtained for comparable separations, reporting the optimum performance achieved in each referred study. All the studies are typically carried out investigating the role of filler loading, feed concentration and operation temperature. However, the general trends are mainly affected by phenomena such as cluster formation, membrane swelling and interaction among permeating species as well as by changes in driving force.

Filler loadings rarely exceeding 5 wt% are used in the recovery of alcohols and phenols from aqueous solutions, where the alcohol concentration is always 5% or less. Permeation fluxes increase as the molecular weight of the alcohol to be recovered rises, independently of filler type and operation temperature. Separation factors range from 10 to 25 in the case of IPA and butanol, whereas they are always less than 5 for ethanol/water solutions (see Table 5).

Depending on the organic molecule recovered from water, a wide range of permeation flux and separation factor values are obtained (see Table 6). Most filler concentrations do not exceed 10 wt.%. Pyridine and ethyl acetate are the organic substances more investigated, for the former Cu_2_O and MoS_2_ are the preferred fillers in Pebax^®^2533 MMMs, for the latter ZSM-5 zeolites. Five times more dilute water solutions are considered in the case of pyridine than ethyl acetate, at comparable temperatures. Operating temperature favourably influences both permeation flux and separation factor in the case of pyridine, while the turbulent flow regime improves performance by just over 5% compared to laminar flow in the ethyl acetate recovery. Particularly important are the enrichment factors achieved by processing very dilute aqueous solutions of toluene, using a low content of NaX zeolite. TiO_2_ was successfully used to process acetone or humic acid water solutions. The high flux reported for the furfural separation [38] could markedly reduce capital investment and equipment footprint. 

Gasoline desulfurization is an interesting application of PV. The nanocomposite membranes investigated for this separation are all in the form of TFN (Table 7). In this membrane structure, the partial intrusion of the Pebax macromolecules within the pores of the support could increase the membrane stability. Quite high fluxes are reported in the revised papers compared to those measured in other PV applications. According to Pan et al., the performance of the HMS(200) filled membrane surpasses the upper bound for the thiophene/n-octane separation [21]. Indeed, the shell of HMS(200) presents pores (size of 0.53 × 0.56 nm) that are between the thiophene (kinetic diameter 0.53 nm) and the n-octane molecular size (kinetic diameter 0.63 nm) [21]. 

The membrane thickness is one of the parameters that affect PV performance. Usually, as the film thickness increases, the permeate flux decreases. This behaviour is also verified using Pebax membranes [72]. Instead, the enrichment factor rises and typically, tends to attain a plateau in thicker films. Thus, depending on the explored range for the membrane thickness, the selectivity can be independent of the selective layer thickness [36]. 

In addition, according to a resistance-in-series model, the boundary layer resistance on the feed side can be predominant for thinner membranes, while the membrane resistance becomes relatively unimportant. Thus, the pervaporation through thin membranes is mainly determined by the hydrodynamic conditions on the feed side. Similar results were reported for the pervaporation of dilute solutions of toluene and phenol through Pebax membranes [87]. Therefore, in order to neglect the liquid boundary layer resistance, a certain thickness is required. On the other hand, thicknesses that are too thin (e.g., less than 2.5 μm) can produce defective films. 

### 5.3. Fuel Cells and Batteries

Fuel cells generate electrical energy by converting the chemical energy of a fuel. This technology does not involve the combustion step, avoiding pollution at their point of use. Accordingly, fuel cells represent an efficient and sustainable energy source for stationary applications as well as for portable and automotive purposes [88]. The membrane within a fuel cell requires correct humidification in order to ensure high ion conductivity and life time. Polymer membranes have been widely used to heat and humidify the fuel streams entering the fuel cell. The fuel cell exhaust is a hot stream of humid air that is directed to one side of a water-permeable membrane while the feed dry gases are sent to the other side of the membrane. A transport of heat and water vapour from the wet stream to the dry stream occurs, avoiding the oxygen cross-over. Compact systems can be produced by adopting humidifier systems equipped with polymeric membranes. In Proton Exchange Membrane Fuel Cells (PEMFCs) the requirements of membrane humidification are increasing, stimulating the fabrication of more performant MMMs.

Membranes based on the neat Pebax^®^1074 demonstrated an efficient capacity to adsorb water vapour, showing very high vapour/air selectivity [89]. Therefore, this copolymer was selected in recent works to prepare MMMs for dehumidification purposes as an alternative to the benchmark Nafion.

MMMs incorporating a clay (montmorillonite, MMT) into Pebax^®^1074 were fabricated by the solvent casting method, in order to obtain a good permeability to water vapour and a low transport rate for air [46]. Poly(oxyalkylene)amine (APOP) was used to modify the MMT to improve its compatibility with the Pebax matrix, without reducing the hydrophilicity of MMT. Surface hydrophilicity and crystallinity of MMMs were enhanced by loading the MMTs and these properties were further boosted by the APOP-MMTs. The vapour/air selectivity was increased by the APOP-modified MMTs, reaching a value of 17 × 10^4^, ca. four times higher than that of pure Pebax^®^ 1074. The vapour permeability was enhanced as well. 

The 2D mesoporous nanosheets of cerium fluoride oxide (F-Ce) and their composite with the IL 1-ethyl-3-methylimidazolium dicyanamide ([Emim][DCA]) were loaded into Pebax^®^1074, attaining improved permeability and selectivity of water vapour [47]. Indeed, the slit-shaped mesoporous structure of the nanosheets facilitates the creation of rapid transport channels for water vapour. The performance of MMMs loaded with IL@F-Ce nanosheets is much compared to the pristine filers. The highest vapour permeability was obtained at a 4 wt% of IL@F-Ce, with a permeability gain of more than two times, while the selectivity increased by 83% compared to the neat Pebax membrane. 

The porous structure of UF membranes facilitated water permeation, resulting in better performance than dense Nafion-based membranes for PEMFCs [90]. Porous humidification membranes were fabricated by combining Pebax^®^3533, polyacrylic acid (PAA) and cellulose nanocrystals (CNCs) in an electrospinning device [48]. CNCs were used to improve the hydrophilicity of the membrane, while PAA was added to increase the spinnability of the material. The electrospun mats were annealed and then repeatedly pressed at high temperatures (120 °C). As a result, the fibres of the mat melted and bonded to each other and transparent fibre membranes of ca. 50 microns were obtained. The optimal content of CNCs was 4.8 wt%, corresponding to the maximum in tensile strength and elongation at break. The air barrier properties of the fibre membranes improved, while the permeability for water vapour decreased.

Table 8 summarizes the properties of these nanocomposites, comparing them to the benchmark Nafion membranes as dehumidifiers for PEMFCs. The porous fibre membranes display a better air barrier behaviour than the Nafion membranes, while their vapour/air selectivity was similar to Nafion 212 and better than Nafion 211 [48]. However, the MMT fillers were more effective in improving the barrier properties to air due to their lamellar configuration that increases the pathway tortuosity [46]. At the same time, the vapour permeability was enhanced due to the increased hydrophilicity imparted by the MMTs [46]. 

The most performing material is obtained by combining Pebax^®^1074 with IL@F-Ce [47]. Therefore, the Pebax^®^1074/IL@F-Ce MMMs are promising as membranes in humidifier devices for PEMFCs.

Recent studies evidenced the positive role of Pebax as a matrix for electrolyte membranes to be applied in ultralong lifespan all-solid-state Li-metal batteries (ASSLBs) [91,92,93]. Solid polymer electrolytes (SPEs) are actively investigated in order to substitute liquid electrolytes producing highly safe electrochemical energy storage. An enhanced Li^+^ conductivity is achieved using the Pebax block copolymer with respect to the homopolymer poly(ethylene oxide) (PEO) and also the mechanical stability is improved. 

Thin dense membranes as heterogeneous nanodomain electrolytes (HNEs) were fabricated by loading lithium bis(trifluoromethanesulfonyl)imide (LiTFSI) in Pebax [91]. The solvent evaporation-induced phase separation forms PEO conductive nanodomains that are interconnected and form ordered transport channels with high ionic conductivity. On the other hand, the PA chains coordinate with the anions of Li salts, enabling fast Li^+^ conduction. A lithium conductivity of 4 × 10^–4^ S cm^–1^ at 60 °C was achieved, while the PEO has an insufficient value of 10^–3^ S cm^–1^ at room temperature. The resulting ASSLBs display superior cycling stability with a capacity retention of 80% after 1560 cycles under the current density of 0.5 C. 

Plasticizer-free composite block copolymer electrolytes with conductive nanodomains were prepared by combining Pebax copolymer as a conductive framework and polyethylene glycol dimethyl ether (PEGDME) [93]. The use of liquid plasticizers to enhance ionic conductivity in SPEs results in a loss of safety. PEG is a regulator that is capable of connecting highly conductive nanodomains. These nanodomains in composite BCEs can be obtained with tunable size and with a homogeneous Li^+^ deposition. The all-solid-state LiFePO_4_/Li cells achieve remarkable electrochemical performance, showing capacity retention of 83% for more than 1350 cycles at 0.5 C (e.g., minor capacity reduction of 0.0127% per cycle). 

Both papers [92,93] report a suppressed Li dendrite growth during repeated cycling due to the matrix mechanical strength.

Nanofibrous membranes were produced by using an electrospinning technique incorporating a natural filler in Pebax [91]. The plasma-treated Bacterial Cellulose Acetate extracted from nata de coco waste was distributed homogeneously in the Pebax nanofibers. The versatile electrospinning technique allowed to produce nanofibrous membranes with high porosity, allowing a suitable electrolyte loading. The ionic conductivity of the electrospun PEBAX/2BCA reaches values of 9 × 10^−3^ S/cm with no substantial shrinkage at 150 °C. Compared to a commercial polypropylene membrane (Celgard2400 separator), the nanofibrous membranes present good wettability while the high porosity results in a large amount of electrolyte absorption and thus in a strong ionic conductivity.

### 5.4. Membrane Filtration for Water Treatment 

Environmental remediation is today urgent and a lot of studies are exploiting nanomaterials to purify wastewater streams according to different processes such as the adsorption of heavy metals and other pollutants (e.g., pharmaceuticals), the pathogen removal and inactivation, the degradation and conversion of toxic species [94]. The use of membranes containing nanomaterials is an advanced wastewater treatment that can be selected to perform selective filtration processes or to combine the filtration with catalytic remediation processes [95,96].

Hydrophobicity is the main issue that determines the membrane fouling, particularly for PES and PSf porous membranes. Therefore, a thin hydrophilic polymeric layer coated on porous hydrophobic supports is an effective approach to deal with membrane fouling, guaranteeing at the same time an adequate water flux. Nanocomposite membranes with antifouling properties were developed as thin-film nanocomposite (TFN) membranes in which a thin layer of Pebax/fillers MMMs is coated on a porous support [42,43,97]. 

Mousavi et al. incorporated multiwalled carbon nanotubes up to 2 wt% after their wrapping with chitosan (CWNTs) and coated PES supports with a very thin mixed matrix layer of Pebax^®^1657/CWNTs [42]. The produced membranes were investigated for dye removal (Malachite green) from water. The chitosan layer was not covalently linked to the CNTs, as confirmed by FT-IR and XRD, and improved the compatibility of the fillers with the Pebax. The acidic conditions of the investigated separation result in positive charges for chitosan, due to the protonation of N-H groups of chitosan and, thus, in the electrostatic repulsion for the positive malachite green. The CWNTs increased the membrane hydrophilicity, improving the antifouling properties. Agglomeration of nanofillers was observed at loadings higher than 1%. 

Membranes for the separation of oil/water emulsions by nanofiltration were obtained by coating a porous PSf support with a thin layer of Pebax loaded with Functionalized Multiwall Carbon Nanotubes (F-MWCNTs, up to 2 wt%) [43]. The morphological analysis did not evidence interface voids or sieve-in-cage morphology. The presence of the F-MWCNTs increased the mechanical resistance, hydrophilicity and thermal stability of the host matrix as well as the oil rejection. A maximum in the permeate flux, as a function of F-MWCNT loading, was observed at 0.5 wt%. Oil rejection shows an upward trend by increasing F-MWCNT loading and was enhanced by using a low transmembrane pressure. 

TFN membranes were prepared by coating a highly porous electrospun nanofibrous scaffold (based on cross-linked polyvinyl alcohol (PVA)) with a hydrophilic barrier top layer that is based on Pebax^®^1074 incorporating surface-oxidised multiwalled carbon nanotubes (MWNTs) up to 10 wt% [97]. The surface of oxidised carbon nanotubes creates additional water channels. Thus, the nanofibrous TFNs present considerably high flux in the treatment of simulated bilge water, containing 1350 ppm of soybean oil and 150 ppm of an emulsifier (DC 193, polysiloxane–polyethylene glycol), keeping the same high rejection ratio (ca. 99.8%). 

TiO_2_ nanotubes were loaded in Pebax 1657 developing dense membranes successfully applied in a UV-assisted desalination system at room temperature [45]. To prevent agglomeration, the titania particles were treated by sonication and then the priming method was used to provide a homogeneous distribution within the polymeric layer. The hydrophilic TiO_2_ nanotubes increased the water flux and the best results were achieved at a TiO_2_ loading of 10 wt%. The membrane swelling and the salt rejection were enhanced upon the TiO_2_ incorporation. The UV irradiation efficiently contributes to reducing the conductivity of the permeate water (ca. ten times, from 206 μS to 21 μS), since the activated TiO_2_ degrades organics and prevents surface cake formation. 

A summary is proposed in Table 9. The permeation flux observed in 2533 Pebax is one order of magnitude higher with respect to the 1657 grade, whereas the rejections are comparable. In the case of MWCNTs, a functionalization prior to the dispersion in the polymer matrix is advisable, differently from titania nanotubes. 

## 6. Other Applications

### 6.1. Adsorption 

**Figure 6:**
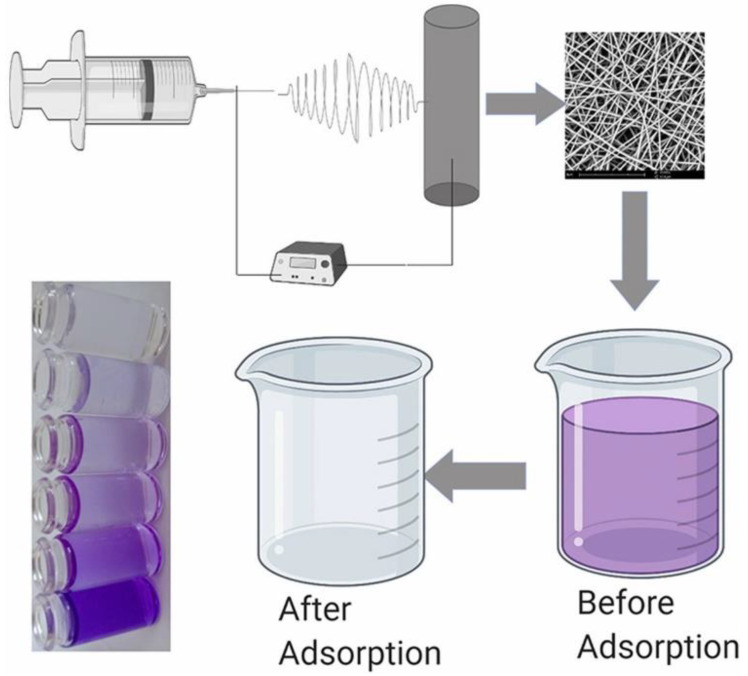
Pebax-based nanocomposite fibrous membranes fabricated by melt electrospinning and applied to dye removal (from ref. [50]).

Nanocomposite membranes for the adsorption of contaminants from wastewater were developed by loading graphene oxide (GO) into Pebax [49,50]. This approach allows to heterogenize nanoparticles that display selective adsorption capacity and antibacterial properties. Zielińska et al. investigated the adsorption of pharmaceuticals (sulfadiazine, amoxicillin and tetracycline) from water [49], while Sarwar et al. studied the removal of cationic dyes (crystal violet) [50]. The membranes developed by Zielińska et al. [49] are prepared by phase inversion. The GO with a higher surface area (0.0290 m^2^) removes up to 6–8% of pharmaceuticals, which is twice the amount adsorbed by the GO nanomaterial having a surface area of 0.0145 m^2^. This performance is below that of a GO suspension since when the nanoparticles are embedded into the matrix, the polymer chains cause pore blocking. Instead, the membranes developed by Sarwar et al. are fibrous samples prepared by the melt electrospinning process (Figure 6). Paraffin liquid was mixed with Pebax 3533 to attach the nanofillers (thickness 10–20 nm and a few microns in length). A concentration of 2% GO was the most effective for increasing surface area (due to the smaller fibre diameters) and hydrophilicity of the fibrous membranes, also providing the fastest adsorption kinetics of the cationic dye from water. The membrane containing 2% GO reached ca. 100% removal efficiency (at a dose of 40 mg/L and pH of 10). Instead, a higher GO concentration was not suitable for the electrospinning process, causing instability [50].

Indeed, despite the majority of the Pebax MMMs being in the form of dense membranes, porous membranes were also produced by the electrospinning process as fibrous networks. 

Electrospinning fabricates polymeric fibres by ejecting a polymer solution under high electric fields. The fibres form highly porous webs combining small pore sizes and a high surface-to-volume ratio that are suited for biomedical devices, water purifying systems and disinfection. Electrospinning allows manipulation of the fibre diameter and microstructure. In addition, the technique is low cost, rapid, relatively easy and can be potentially applied to diverse materials, including insoluble nanoparticles blended in the polymer solution [95,98]. Electrospun matrices can expose a higher amount of highly porous nanoparticles for adsorption, supporting them with robust structures and allowing to maintain their functionality. In addition, the fouling phenomena are less relevant due to the high porosity that results in high permeability.

Sarwar et al. studied the process using neat Pebax 3533, comparing melt electrospinning to solution electrospinning (using butanol as solvent) [99]. Hydrophobic membranes were obtained, without altering the polymer properties. The melt process produced larger fibres (ca. 2 microns in diameter) than those produced using a solvent (ca. 0.4 microns). 

Fibrous Pebax nanocomposite membranes were prepared by combining the extrusion process to prepare filaments and melt electrospinning to convert them into a fibrous matrix [44]. Graphene nanoplatelets were added to Pebax up to 0.4 wt%, while paraffin liquid (0.2 wt%) was used as an additive to improve the polymer/filler adhesion. A uniform nanofiller dispersion was observed in the nanocomposite PEBA filaments. The membranes obtained at the lowest speeds can be used in the cleanup of oil spills due to the small inter-fibre pores. The optimum graphene concentration in the polymer matrix was 0.3 wt%, corresponding to an increased polymer crystallinity (+58% in fibrous membranes compared with pure PEBA) that provided the maximum adsorption capacity, while at higher concentrations of graphene, aggregates were found. 

The melt electrospinning described in [81,84] to convert the Pebax MMM filaments into porous membranes is an environmental-friendly additive manufacturing technology. Three-dimensional printing, also referred to as Additive Manufacturing (AM), is a rather novel technology with many interesting aspects such as minimized waste, print on demand, etc. [100,101]. Recently, 3D manufacturing was extended to the membrane preparation [102,103,104] as well as to the manufacturing of the spacers commonly included in the membrane modules used for liquid filtration [105,106]. 

### 6.2. Electromagnetic Shielding/Sensors/Detectors

Electromagnetic shielding is essential to ensure protection from electromagnetic pollution, e.g., from the radio frequency radiation of electronic devices [107]. Excellent electrical conductivity is a key requirement for the shields. Therefore, metal-based materials are usually used for electromagnetic interference (EMI) shielding, but they are penalized by poor corrosion resistance, high density and secondary radiation. Instead, polymers are lightweight and possess easy processing and superior flexibility [108]. Nanocomposites are an effective solution for high-performance EMI shielding materials, being lightweight and resistant to corrosion and combining good processability with adjustable conductivity. 

Flexible and lightweight porous EMI shields were obtained as Pebax/MWCNT nanocomposite films applying a novel biaxial stretching-assisted microcellular foaming technology [52,53]. The MMMs having sub-microcellular and nanocellular structures display electrical conductivities almost two orders of magnitude higher than nanocomposite films having a microcellular or dense structure. The EMI shielding capability was found to increase in the samples with smaller cell sizes. Thus, the cellular MMMs showed better total shielding effectiveness than the unfoamed pristine MMM samples (41 dB vs. 26 dB). 

Another study analysed the effect of the Peba composition on the electrical conductivity of Peba/MWCNT nanocomposites [53]. Indeed, the loading of conductive nanofillers as CNTs, which usually act as nucleating agents in semicrystalline polymers, produces crystals on the filler surface that hinder the creation of electrical percolation pathways. CNTs can nucleate the crystallization of the PA block of Peba. Thus, a lower amount of the PA block (e.g., 20 wt% in Pebax 2533) resulted in higher electrical conductivity and better EMI shielding performances. The best solution was found using the 2080 Pebax grade in which the PE block is the ionically conductive polyethylene oxide that effectively reduced the hampering effect of PA crystals on electrical percolation. Therefore, a high conductivity was combined with an enhanced amide dipole moment due to the high PA content (50 wt% in Pebax 2080). This feature assists the electromagnetic wave absorption that becomes the dominant mechanism for EMI shielding (shielding effectiveness of ca. 15 dB at 5% of CNTs in Pebax 2080). 

EMI shields were produced by a facile melt blending followed by compression moulding, obtaining flexible Pebax films incorporating graphene [51]. The Pebax/graphene composites display an electrically conductive percolation threshold of 1.75 vol% graphene, reaching a shielding effectiveness of 31 dB at 20 wt% (i.e., 8.9 vol%) graphene. A negative pressure effect of resistance was observed for the Pebax/graphene composites: their electrical resistance gradually decreases as a response to increasing outer pressure stimulation. This behaviour was attributed to the creation of more conductive pathways when the distance between adjacent graphene sheets is reduced [108]. These elastomer films could be used as pressure sensors. 

Indeed, membranes are key components of different types of sensors. Their role moves from simple mechanical support, a portion of the transduction mechanism to a filter of pathogens or an engineered reservoir of active proteins [109].

A hydrophilic Pebax alone was investigated as a polymeric resistive humidity sensor due to its good chemical stability combined with very high water adsorption and high melting point [110]. Pebax thin films were coated on alumina substrates with gold comb electrodes by spinning, using a phenol solution of the copolymer. The sensors show no hysteresis in the humidity-impedance plot, with slow response times for humidification and very quick responses for desiccation compared with conventional sensors. In addition, good reproducibility and long-term stability were observed.

Humidity sensors were produced by electrospinning, loading LiCl·H_2_O into Pebax^®^2533 [57]. At a LiCl·H_2_O content of 44.4 wt%, the sensor showed the best sensing performance since its impedance changed nearly four orders of magnitude when the relative humidity changed from 11% to 95% RH, with negligible hysteresis.

A monolithic and flexible sensor for indoor air monitoring was produced by inserting a photoactive TiO_2_ sensing film between a flexible polyethylene terephthalate substrate and a Pebax MMM (Pebax^®^1657 filled with ZIF-7 nanoparticles) as an overlayer [54]. This chemiresistive sensor, used under UV illumination to promote photoactivation, is ultrasensitive, detecting ppb-level of formaldehyde, a harmful indoor pollutant, at room temperature. Indeed, while the TiO_2_ film alone showed non-discriminative gas responses, the Pebax MMM overlayer allowed the removal of the interference of other indoor pollutants (e.g., ethanol) by molecular sieving. 

Flexible composites based on Pebax were applied in piezoresistive sensors that detect electrical signals upon an external pressure/strain stimulus [111]. They find applications in health monitoring, wearable devices and in motion detection requiring good reproducibility, high flexibility, sensitivity and processability. Microcellular Pebax beads obtained by supercritical CO_2_ foaming were dip coated into a GO suspension; a subsequent compression moulding produced 3D conductive channels between the contiguous beads. The in situ reduction leads to reduced graphene oxide (RGO@Pebax) composites that are lightweight (0.2 g/cm^3^) and present electrically conductive paths and high flexibility (up to 50% compressibility). The produced sensors combined a bigger contact area due to the 3D conductive channels with “disconnect-connect” transition of microcracks. 

The Pebax flexibility was exploited to incorporate a high amount of Pd without embrittlement of the material, developing sensing materials with specific nanoparticle locations and properties. Nanocomposite films containing Pd amount up to 30 wt% (corresponding to 3.5 vol%) were prepared by using an in situ generation route from the palladium acetate precursor and through a solution casting process [112]. The crystalline Pd nanoparticles were located in the Pebax soft phase, resulting in conductive materials for Pd amounts as low as 3.5 vol% (electrical conductivity = 5.1 × 10^−6^ S/cm). In addition to the flexibility, mechanical stability and hydrogen adsorption enable the use of the developed nanocomposites as sensing materials. 

### 6.3. Biomedical Applications

Flexible films composed of Pebax copolymers filled with nanoparticles were developed for different biomedical applications such as the prevention of microbial contamination [55]. Antimicrobial materials are actively investigated all over the world since the occurrence of infectious diseases within healthcare facilities exacerbates patient illness and, in some cases, can be lethal. In this respect, antimicrobial surfaces can reduce microbial contamination and transmission allowing to control healthcare infections. 

Different radiopaque fillers (i.e., dense metal or ceramic powders) were loaded within biomedical Pebax^®^ grades (Pebax 7233 SA01 MED, Pebax 5533 SA01 MED and Pebax 2533 SA01 MED) using a co-rotating twin-screw extruder [113]. The developed materials are suited for producing catheters, permitting their localization and/or accurate positioning inside the body (e.g., using X-ray imaging). Indeed, Pebax is available in a wide range of durometers and flexibilities, allowing the production of catheters with progressive stiffness along the tube length. In addition, the possibility to deform the Pebax tubes permit their use at the distal end, avoiding trauma to the blood vessels.

Pebax/MMT nanocomposites, obtained by melt compounding using organically modified nanoclays, showed improved ultimate tensile strength and strain at break, thus allowing their use for producing angioplasty balloons [114]. 

Scaffolds based on Pebax and layered double hydroxide (LDH) biomaterials were developed as wound dressings, investigating the in vitro drug release kinetics and cytotoxicity [56]. The LDH lamellae containing Mg^2+^ or Zn^2+^, Fe^3+^ and Al^3+^ cations were intercalated with chloride anions or naproxenate (NAP) anions. NAP LDH improved the mechanical stability of the membranes as a consequence of a better LDH–polymer interaction. Advantages in terms of cell viability were observed for the Mg-LDH membranes with respect to powdered samples. Pebax containing Mg-NAP intercalates showed the best global performances: faster NAP release, greater mechanical resistance and improved cell viability compared to the pure components (i.e., Pebax, NAP and LDH).

Hot-melt extrusion (HME), widely used industrially, was adopted to produce light-activated antimicrobial polymer surfaces. This method can be easily implemented in large-scale manufacturing. The nanocomposites were based on a range of medically relevant polymers, including different Pebax grades, loaded with a photosensitiser (toluidine blue O, TBO) [55]. Upon light exposure, the photosensitiser generates reactive oxygen species that result in antimicrobial properties. Singlet oxygen can effectively eradicate bacteria, bacterial spores, fungi and viruses by oxidising pathogenic cells leading to their death. Pebax^®^ 2533 was the most effective polymer against bacterial strains of *S. aureus*. The Pebax^®^ 2533 extrudates incorporating 0.1 wt% TBO showed a significant resistance to bacterial colonies upon exposure to ambient light conditions. The viable bacterial adherence for different pathogens frequently encountered in hospitals, such as *Staphylococcus aureus*, *Staphylococcus epidermidis*, *Acinetobacter baumannii* and *Escherichia coli*, was reduced by >99.9%. 

Pebax^®^ Rnew was successfully combined with Imidazolium-based ILs producing flexible films with antibacterial behaviour by solution casting [59]. Pebax^®^Rnew is a plasticizer-free TPE copolymer that comprises polyamide 11 domains obtained from castor oil. Thus, the copolymer is up to 90% derived from renewable sources. Two ILs (1-hexadecyl-3-methylimidazolium dimethyl-5-sulfoisophthalate [Hdmim][DMSIP], IL1 and 1-octyloximethyl-3-methylimidazolium hexafluorophosphate [OOMmim][PF_6_], IL2) were loaded up to 5 wt%. The films containing [Hdmim][DMSIP] that present a long alkyl chain in the cation provided an interesting antimicrobial activity, combining hydrophilicity, permeability and thermal stability. Instead, the smaller IL2 is readily released from the films without altering the hydrophobic nature of the neat polymer. 

Sole et al. produced breathable membranes by blending Pebax MH 1657 with different organic molecules [60,61,62]. Dense films were prepared via the solution casting method. Mercaptoethanol (ME) improved the mechanical properties by introducing crosslinks within the polymer matrix and 30% was its most effective concentration [113]. Sulphur–Chlorine Bifunctional Diol (SCBD) was used to impart antimicrobial properties to the films exploiting the concurrent presence of sulphur and chlorine [56]. The PEBA/SCBD membranes show an antimicrobial behaviour even at 2.5% SCBD (up to 99.99% bacterial reduction) while using chloropropane diol (CPD) excellent antimicrobial properties were observed at higher concentrations (≥10% [56]). The water vapour transmission rate (WVTR) in the CPD-loaded membranes increased from 1500 g/(m^2^ day) to 2350 g/(m^2^ day) at 40% CPD [56]. However, at the high modifier content, thermal and mechanical properties are degraded [56]. Instead, the films comprising 2.5% SCBD possess acceptable WVTR (1530 g/m^2^/day) and permeability (WVP = 16.4 g μm/m^2^ day Pa) to be used as breathable films for environmental (wastewater treatment, helium separation) or biomedical (breathing masks, bandages, surgical gloves and gowns) applications [61].

Antibacterial materials were obtained as Pebax^®^ nanofibers containing Ag nanoparticles produced by electrospinning [58]. In particular, Pebax was electrospun with AgNO_3_ and the obtained samples were treated with UV light to reduce the silver ions to metallic Ag nanoparticles. The Ag nanoparticles (15 nm in size) were uniformly distributed in the Pebax fibres and decreased the polymer crystallinity as well as the onset temperature for decomposition. A very low amount of Ag salt was used (0.05–0.25‰) by Liang et al. [58]. No antibacterial activity was observed for neat Pebax, while at an ultralow concentration of AgNO_3_ in Pebax^®^ (0.15‰) the inhibition rate was >99.9% and the antimicrobial activity against *Escherichia coli* and *Staphylococcus aureus* was 5.8 and 5.6, respectively [58]. This Ag content is much lower than that of other Ag/polymer composites (typically larger than 1 wt%). Potential applications for the antibacterial Ag/Pebax material are biomedical materials, sports apparatus and laminating films.

### 6.4. Packaging Applications

Pebax-based nanocomposites were tested for the packaging sector. One of the requirements for this application is stability under electron beam irradiation since this is a common method to sterilise pharmaceutical packaging products and medical devices. Virgin Pebax, exposed to various electron beam and gamma irradiation doses, presented branching and chain scission [115]. The radiation stability to electron beam irradiation of films based on Pebax (6333 grade) and two stabilisers (phenolic antioxidant and light stabiliser) was studied [116]. Different packaging and processing conditions (vacuum, dry ice) were applied to reduce or exclude chain scission, crosslinking and oxidative reactions. Virgin Pebax in non-vacuum packaging immersed in dry ice provided superior resistance to radiation (20.9% improvement) compared to the traditional packaging method. 

Thin transparent and breathable films were prepared for food packaging applications by the solvent casting method combining Pebax^®^ MH1657 with polyethylene glycol dimethyl ether (PEGDME) and/or hydroxyl-terminated polyethylene glycol (PEGOH) [63]. PEG decreases the membrane crystallinity. Pebax^®^ and PEG were totally miscible for a PEG amount less than 30 wt%. This study demonstrated that a wide range of films could be prepared with tailorable mechanical and gas transport properties, capable to prolong the shelf-life of highly breathable fresh products.

**Figure 7:**
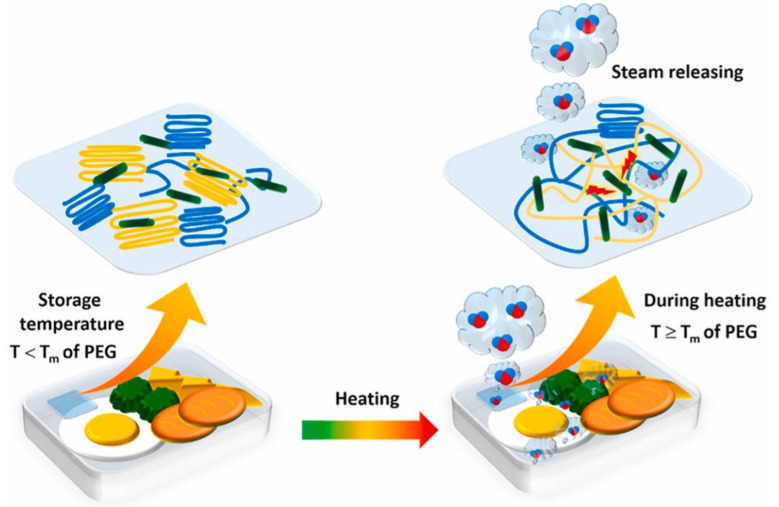
Nanocomposite films based on Pebax and HNTs as self-venting packaging materials (from ref. [64]).

Self-venting packaging materials were obtained by adding halloysite nanotubes (HNTs) to a matrix composed of Pebax and polyethylene glycol (PEG) [64]. The films evidenced a temperature-responsive behaviour due to the PEG presence. The addition of HNTs, by disrupting the intermolecular (H-bonding) interactions between the polymer chains, improved the water diffusion rate and decreased the phase transition temperature of PEG, while the water sorption was not altered. The mechanical strength of the films was preserved by the HNTs that supported the PEG in their hollow tubular structure when the nanocomposite film became weak and swollen. The films with a low loading of HNTs (5–10%) did not present cracks upon the thermal cycles, releasing steam at a temperature close to that for the phase transition of PEG. Thus, they could be applied in the rewarming of packaged foods or sterilising biomedical products under safe conditions (Figure 7).

### 6.5. Further Uses of Pebax

Different studies investigated the behaviour of Pebax nanocomposite structures via a comprehensive characterization. 

Nanocomposites based on Pebax and graphene were examined by analysing their crystallization kinetics during the extrusion process with and without supercritical fluid carbon dioxide (scCO_2_) [117]. The scCO_2_ processing leads to a reduced crystallization rate. The decreased crystallite size (from 4.49 nm for neat Pebax to 3.48 nm for PebaxSCF) indicates that scCO_2_ processing involves a rearrangement of polymer chains to kinetically more favourable configurations, improving homogeneity between amorphous and crystalline parts of the Pebax copolymer. At the same time, graphene exfoliation is promoted. 

Among the most recent trends, wood/polymer nanocomposites, materials for protection against chemical warfare agents (CWAs) and materials for microelectronic cooling were developed as detailed below.

Sliwa et al. [118] studied the thermal stability of a new family of wood polymer composites based on hydrophilic Pebax^®^ copolymers that can interact with the wood fibres. The thermal stability of the composites displayed a noteworthy improvement under air atmosphere. Therefore, the loading of wood fibres in Pebax^®^ delays the thermo-oxidation in air due to the formation of char residue in the earlier stages of degradation. Tensile strength, modulus and impact strength were significantly increased upon the addition of nanolignocellulose (LCNFs, up to 5 wt%) to Pebax by compression moulding using a hot press [119]. These nanocomposites combine biodegradability with improved water absorption and thickness swelling.

Pebax was successfully adopted to improve the dispersion of boron nitride nanosheets in an epoxy resin [120]. Pebax functionalized h-BNNSs (P-BNNSs), obtained by mechanical exfoliation and in situ modification process, enhanced the thermal conductivity and antiwear performance of the epoxy matrix. A multinetwork structure was obtained at a low P-BNNS loading (≤6 wt%) thanks to the hierarchical assembly of P-BNNSs during the curing process of the EP matrix. This structure permits a simultaneous vertical and horizontal heat diffusion, resulting in quasi-isotropic thermal conductive materials (through-plane thermal conductivity of 3.9 W/(m·K) and in-plane thermal conductivity of 2.9 W/(m·K)), allowing heat dissipation in high-density integration systems or in high frequency printed circuits. 

The adhesive properties of Pebax were exploited to attach MOF particles (UiO-66-NH_2_ and HKUST-1) to an inert PP substrate in order to develop materials for personal protective equipment against toxic chemicals [65]. The performance of the composites against the chemical warfare agent simulant 2-chloroethyl ethyl sulfide (CEES) depends on the type of Pebax used. On the other hand, the underlying mat with higher density provides increased protection against CEES. Therefore, combining the hydrophobic Pebax 2533 and a higher fibre density led to better barrier properties. The best material offered ~250 min protection (to a permeation rate of 0.1 μg/min/cm^2^) while the protection of activated carbon fibres is ∼150 min. Interestingly, the use of Pebax as an adhesive stabilises HKUST-1 to moisture. 

Finally, an interesting study proposed to use Pebax 2533, dissolved in isopropanol, as a template for the solvothermal preparation of monodisperse Mn_3_O_4_ nanoparticles [121]. A larger Pebax concentration reduced the size of the nanoparticles (9–32 nm) leading to small particles with a greater capacitance when applied in a supercapacitor (e.g., 217.5 F/g at a current density of 0.5 A/g).

## 7. Conclusions

Nanocomposites based on commercial thermoplastic elastomers referred to as Pebax were recently developed and tested in the preparation of membranes for separating gases and for wastewater treatment. Neat Pebax does not offer excellent performance in separating specific species (e.g., in olefin/paraffin or in pervaporation), while specific material combinations result in significantly better separation performance. The majority of the revised studies on membranes are related to the pervaporation process, investigating the influence of operating conditions such as feed temperature and concentration on process performance. Depending on the Pebax copolymer grade, both hydrophilic and hydrophobic membranes can be produced. However, the most used Pebax grade is the hydrophobic 2533 grade owing to the high amount of flexible polyether block. 

The design of the membrane structure and the choice of materials are key factors for achieving optimal performance. Supported membranes are the ideal configuration for pervaporation or water treatment owing to the increased flow rates obtained by thinner separative layers. Supported hollow fibres are also produced for pervaporation applications. On the other hand, the rubbery Pebax matrix well accommodates advanced materials capable of providing better separation properties. Therefore, several fillers with distinctive features are successfully dispersed in Pebax matrices, including metal oxide nanoparticles, carbon-based materials (nanotubes, graphene, graphene oxide), clays, mesoporous silica, zeolites, Halloysite nanotubes and MOFs. 

The effects of the fillers on the morphology and separation properties of the resulting membranes were systematically studied, revealing that the loading amount has to be optimized in nanocomposite development. Indeed, low amounts of particles are capable to disrupt the intrinsic arrangement of the polymer chains, thus enhancing the available free volume in the matrix. Instead, higher loadings can induce a partial polymer chain “rigidification”, but inevitably result in the formation of agglomerates that typically cause a delay in the material performance. However, detrimental phenomena at the interface of heterogeneous phases are reduced by the use of compatibilising agents, capable of facilitating the incorporation of the filler within the host matrix (e.g., ionic liquids) or by fillers having organic moieties (e.g., covalent organic frameworks).

Novel strategies to design high-performance nanocomposite membranes include the manipulation of the filler’s morphology, leading to 2D configurations (e.g., leaf-like MOFs) or the use of fillers with a hierarchical structure (e.g., in gasoline desulfurization by PV) or with multifunctional properties (e.g., graphene-based materials). Among the different fillers investigated, those having porous structures introduce a further degree of freedom in the separation due to the additional molecular sieve mechanism. The extensive investigation of the microstructure-performance relationships of the produced MMMs explains the fundamental role of the filler morphology. 

Selected additives are capable to improve membrane performance with respect to benchmark materials (e.g., Nafion for fuel cell humidification) and reduce the swelling phenomena in membrane samples, increasing separation properties and physicochemical stability over time. In this respect, fillers shaped as 2D nanosheets proved particularly effective in improving the permeation and separation capability of the neat copolymers. Thus, membranes incorporating mesoporous nanosheets demonstrate considerable potential for industrial-scale application in both dehydration and humidification. 

This holistic review considers also the nanocomposites developed for a wide range of sensitive applications including electrochemical shielding, sensing devices, packaging films, antimicrobial materials, breathing tissues and biomedical devices such as wound dressings. Nanocomposite films having a porous structure were developed by electrospinning and successfully applied to adsorption processes or sensing. Flexible self-supported structures can be obtained for those applications that do not involve molecular-level separations. Indeed, Pebax copolymers present a tailorable combination of flexibility and partial rigidity of the matrix due to semicrystalline rigid polyamide domains in a continuous soft region of polyether that can be exploited in order to produce unprecedented materials, constructing highly conductive nanodomains for batteries or obtaining the filler segregation only in one copolymer phase. The chemistry of the two blocks permits selective interactions with the additives, thus resulting in reduced leaching of liquid additives (e.g., ionic liquids) as well as excellent compatibility with solid organic and inorganic fillers. 

This work aims to inspire and motivate the development of nanocomposite membranes and materials also in novel applications covering the fabrication of membranes for high-performance lithium-ion batteries, films for electromagnetic shielding and protection against chemical warfare agents and materials for microelectronic cooling. Alternative uses of Pebax are as filler coating agents for improving the exfoliation of inorganic fillers within an epoxy matrix and as glue to assist the deposition of fillers on inert supports.

The excellent progress achieved in recent years enables Pebax-based nanocomposites to become a versatile platform, endowing accessible approaches for molecular separation and sensing as well as for the rapidly growing additive manufacturing industry.

## Figures and Tables

**Table 1:** Composition of commonly adopted Pebax grades.

Grade	Polyamide (PA)		Polyether (PE)	
	Type	Amount (wt%)	Type	Amount (wt%)
5513	PA6	60	PTMO	40
2080	PA12	50	PEO	50
1074	PA12	45	PEO	55
1657	PA6	40	PEO	60
3533	PA12	30	PTMO	70
2533	PA12	20	PTMO	80

**Table 2:** Fillers utilized for developing membranes for separation purposes.

Pebax Grade	Filler Type	Loading (wt%)	Separation	Ref.
**Gas Separation (Olefin/Paraffin)**				
2533 on a poly(vinylidene fluoride) (PVDF) support	AgBF_4_	20–80	Ethylene/ethane Propylene/propane	[11]
2533 on a polysulfone (PSf) support	AgBF_4_/Al(NO_3_)_3_ (1/0.1 mol/mol)	9.7 (AgBF_4_)	Propylene/propane	[12]
1657 on a PSf support	AgBF_4_/Al(NO_3_)_3_ (1/0.1 mol/mol)	17 (AgBF_4_)	Propylene/propane	[13]
1657 on a PSf support	Silver nanoparticle/7,7,8,8-tetracyanoquinodimethane (AgNPs/TCNQ)	87	Propylene/propane	[14]
5513 on a PSf support	AgBF_4_/[BMIM][BF_4_]	10 (AgBF_4_)	Propylene/propane	[15]
2533	nano-zeolite (NaY or NaA)	3–10	Ethylene/propylene	[16]
1657	Ionic Liquids (ILs)	20–60	Recovery of hydrofluorocarbons (HFCs) and hydrofluoroolefins (HFO) from exhausted refrigerant mixtures	[17]
**Pervaporation**				
2533 on a PDMS support	TiO_2_	up to 1.5	Recovery of acetone from wastewater	[18]
2533 on a PSf-PES blend support	TiO_2_ nanoparticles	0.01–0.05% in the coating solution	Removal of humic acid from water	[19]
2533	NaX nano-zeolite	2	Toluene from aqueous solutions	[20]
2533	Hollow monocrystalline silicalite-1 (HMS) (200 nm and 350 nm)	10–50	Thiophene/n-octane (gasoline desulfurization)	[21]
2533	ZSM-5 (3–5 micron)	10–40	Ethyl acetate from water	[22]
2533 on a polyethersulfone (PES) support	ZSM-5 (35 nm)	5–15	Ethyl acetate from water	[23]
2533 on a PES support	ZSM-5 (35 nm) and [Hmim][PF_6_]	1% ZSM-5, 2.5% IL	Ethyl acetate from water	[24]
2533 on a PVDF support	Organophilic zeolitic imidazolate frameworks (ZIF-71)	10–25	Recovery of biobutanol from acetone–butanol–ethanol (ABE) broth	[25]
2533 on a PVDF support	Zn(BDC)(TED)_0.5_	up to 20	Recovery of biobutanol from acetone–butanol–ethanol (ABE) broth	[26]
2533 on a ceramic support Hollow fibre	MAF-6	up to 20	Ethanol from water	[27]
2533 on a PVDF support	ILTf_2_N@MIL-101	up to 10	Ethyl acetate from water	[28]
2533	Mesoporous molecular sieve MCM-41 modified with [EVIM][Tf_2_N] (IL1)	2–10	Butanol from water	[29]
2533	Mesoporous molecular sieve MCM-41 modified with [OMPY][Tf_2_N] (IL2)	2–10	Butanol from water	[29]
2533	Polyhedral oligomeric silsesquioxane (POSS)	2–10	Ethanol from water	[30]
2533	Cu_2_O nanocrystals	up to 15	Recovery of Pyridine from water	[31]
2533 on a PSf support	MoS_2_ Two-dimensional nanosheets	up to 6	Gasoline desulfurization (Thiophene/n-octane)	[32]
2533	MoS_2_ Two-dimensional nanosheets	up to 20	Recovery of Pyridine from a dilute solution	[33]
2533	Graphene nanosheets (thickness of 2–18 nm and less than 32 layers)	up to 2	Recovery of isopropanol from aqueous solution	[34]
2533 on a PSf support	Graphene nanosheets with a dopamine coating and Ag nanoparticle loading (Ag-PDA/GNS)	up to 8	Gasoline desulfurization (Thiophene/n-octane)	[35]
1657 on a PSf support	Graphene Oxide (GO)	0.05–0.5	IPA dehydration	[36]
2533 on a PVDF support	Anisotropic ZIF-L nanosheets (ZLNs)	up to 8	Phenol from water	[37]
2533 on a PVDF support	Metal–organic framework MIL-53(Al)	up to 20	Furfural from water	[38]
2533 on a PVDF support	Co-UMOFNs	7.5–40	Phenol from water	[39]
2533	4-(trifluoromethyl)-N-(pyridine-2-yl) benzamide (denoted as F1)	2.5–10	Ethanol/water	[40]
2533	4-(dimethylamino)-N-(pyridine-2-yl) benzamide (denoted as F2)	2.5–10	Ethanol/water	[40]
2533 on a PSf support	Ag-loaded covalent organic frameworks (Ag^+^@COFs)	3–15	Gasoline desulfurization	[41]
**Water treatment/desalination**				
1657 on an ultraporous PES support	Chitosan-wrapped multiwalled carbon nanotubes (CWNTs)	up to 2	Dye removal from water	[42]
2533 on a PSf support	Functionalized multiwall carbon nanotubes (F-MWCNTs)	up to 2	Nanofiltration of oil/water emulsion	[43]
3533	Graphene (thickness 10–20 nm, a few micrometers in length)	0.05–0.4	Cleanup of oil spills	[44]
1657	TiO_2_ nanotubes	2.5–10	UV-assisted desalination of seawater	[45]
**Humidifier membrane in PEMFCs**				
1074	APOP-MMT	up to 10.7	Water vapour/air	[46]
1074	2D mesoporous nanosheets of cerium fluoride oxide (F-Ce) and their composite with the IL [Emim][DCA] (IL@F-Ce)	up to 10	Water vapour/air	[47]
3533	Cellulose nanocrystals (CNCs) and polyacrylic acid (PAA)	up to 9.1	Water vapour/air	[48]

**Table 3:** Fillers utilized for developing Pebax-based nanocomposites as multifunctional materials.

Pebax Grade	Filler Type	Loading (wt%)	Application	Ref.
2533	GO	5	Removal of pharmaceuticals from wastewater by adsorption	[49]
3533	GO on PEBA melt electrospun fibre	0.5–2.0	Removal of cationic dye from wastewater by adsorption	[50]
2533	Graphene (surface area of 50–200 m^2^ g^−1^, thickness of 1–3 layers)	1–20	Electromagnetic shielding	[51]
7233	MWCNT (outer diameters from 10 to 20 nm, average length of 50 μm)	5	Electromagnetic shielding	[52]
4533 2533 2080	MWCNT (average diameter of 9.5 nm, average length of 1.5 μm)	up to 15	Electromagnetic shielding	[53]
1657	ZIF-7	2.5–40	Sensors	[54]
2533 7033 7233	Photosensitiser, toluidine blue O (TBO)	up to 0.1	Photodynamic antimicrobial materials	[55]
2533	Layered double hydroxides (LDH)	up to 13	Scaffolds as wound dressings	[56]
2533	LiCl·H_2_O	6.7–44.4	Nanofibers as antibacterial materials	[57]
2533	AgNO_3_	0.05–0.25‰	Nanofibers as antibacterial materials	[58]
Rnew 25R53	ILs ([Hdmim][DMSIP], [OOMmim][PF_6_])	up to 5	Antimicrobial films	[59]
1657	Mercaptoethanol (ME)	10–40	Breathable films	[60]
1657	Sulphur–Chlorine Bifunctional Diol (SCBD)	up to 10	Breathable films	[61]
1657	Chloropropane diol (CPD)	10–40	Breathable and Antimicrobial films	[62]
1657	Polyethylene glycol dimethyl ether (PEGDME) and/or hydroxyl-terminated polyethylene glycol (PEGOH)	up to 50	Breathable films for packaging	[63]
1657 and PEG	Halloysite nanotubes (HNTs)	up to 15	Films for packaging	[64]
2533 on PP mat	UiO-66-NH_2_ and HKUST-1	up to 40	Materials for personal protective equipment against toxic chemicals	[65]

**Table 4:** Performance of Pebax-based MMMs in the olefin/paraffin separation.

Pebax Grade	Filler Type	Loading (wt%)	Separation	Permeation Flux	Selectivity	Ref.
2533 on a on a poly(vinylidene fluoride) (PVDF) support	AgBF_4_	67	Ethylene/ethane	21 GPU	210	[11]
2533 on a PVDF support	AgBF_4_	80	Ethylene/ethane (mixed gas, 65% C_2_H_4_, 35% C_2_H_6_)	79 GPU	33	[11]
				16 GPU (aged 9 months)	2.5	
2533 on a PVDF support	AgBF_4_ using H_2_O_2_/HBF_4_ in the dope	80	Ethylene/ethane (mixed gas, 65% C_2_H_4_, 35% C_2_H_6_)	49 GPU	35	[11]
				29 GPU (aged 9 months)	39	
2533 on a PVDF support	AgBF_4_	40	Propylene/propane	10 GPU	30	[11]
2533 on a PSf support	AgBF_4_/Al(NO_3_)_3_ (1/0.1 mol/mol)	9.7 (AgBF_4_)	Propylene/propane (mixed gas, 50:50)	10 GPU	5	[12]
1657 on a PSf support	AgBF_4_/Al(NO_3_)_3_ (1/0.1 mol/mol)	17 (AgBF_4_)	Propylene/propane (mixed gas, 50:50)	22.5 GPU	8.8	[13]
1657 on a PSf support	AgNPs/TCNQ	87	Propylene/propane (mixed gas, 50:50)	10.2 GPU	12.7	[14]
5513 on a PSf support	AgBF_4_/ [Bmim][BF_4_]	10 (AgBF_4_)	Propylene/propane (mixed gas, 50:50)	12.3 GPU	3	[15]
2533	NaY	6	Propylene/ethylene (mixed gas, 80:20)	262 Barrer	13.1	[16]
1657	-	-	HFC/HFO mixture, R454B (83% R32, 17% R1234yf)	197 Barrer (R32)	8.0	[17,21]
1657	IL [C_2_mim][BF_4_]	40	HFC/HFO mixture, R454B (83% R32, 17% R1234yf)	300 Barrer (R32)	15	[17]

1 GPU = 1 × 10^−6^ cm^3^(STP)/(cm^2^ s cmHg), measure unit for gas permeance; 1 Barrer = 1 × 10^−10^ cm^3^(STP) cm/(cm^2^ s cmHg), measure unit for gas permeability.

**Table 5:** Pervaporation performance of Pebax-based MMMs for separating alcohols or phenol from aqueous solutions.

Pebax Grade	Filler Type	Loading (wt%)	Separation	T (°C)	Feed Conc.	Permeation Flux (g/(m^2^ h))	Separation Factor (SF)	Ref.
2533	-	-	Ethanol from water	23	5 wt% ethanol	118 (thickness 30 micron)	2.5	[72]
2533 on a ceramic support hollow fibre	MAF-6	7.5	Ethanol from water	60	5 wt% ethanol	4446	5.6	[27]
2533	POSS (AL0136)	2	Ethanol from water	Room T	5 wt% ethanol	183.5	4.6	[30]
2533	POSS (SO1440)	2	Ethanol from water	Room T	5 wt% ethanol	125.8	4.1	[30]
2533	4-(trifluoromethyl)-N-(pyridine-2-yl)benzamide (F1)	2.5	Ethanol from water	60	5 wt% ethanol	199	4.6 PSI = 916 g/(m^2^ h)	[40]
2533	4-(dimethylamino)-N-(pyridine-2-yl)benzamide (F2)	10	Ethanol from water	60	5 wt% ethanol	173	4.6 PSI = 797 g/(m^2^ h)	[40]
3533	-	-	IPA from water	30	10 wt% isopropanol	300	3.5 PSI = 750 g/(m^2^ h)	[83]
1074	-	-	IPA from water	50	10 wt% isopropanol	260	2.4 PSI = 364 g/(m^2^ h)	[84]
2533	Graphene	1.5	IPA from water	50	4 wt% isopropanol	842; Isopropanol: 249	10.0 PSI = 7620 g/(m^2^ h)	[34]
2533	-	-	Butanol from water	23	5 wt% butanol	179 (thickness 30 micron)	5.9	[72]
2533	Mesoporous molecular sieve MCM-41 modified with [OMPY][Tf_2_N] (IL2)	5	Butanol from water	35	2.5 wt% butanol	422	25	[29]
2533	Mesoporous molecular sieve MCM-41 modified with [EVIM][Tf_2_N] (IL1)	5	Butanol from water	35	2.5 wt% butanol	420	23	[29]
2533 on a PVDF support	ZIF-71 particles (average size of 1 µm)	20	Biobutanol from acetone–butanol–ethanol (ABE) broth	37	0.6 wt% acetone, 1.2 wt% butanol and 0.2 wt% ethanol	Total flux: 520.2	n-butanol SF: 18.8 PSI = 9780 g/(m^2^ h)	[25]
2533 on a PVDF support	Zn(BDC)(TED)_0.5_	20	Biobutanol from acetone–butanol–ethanol (ABE) broth	40	0.6 wt% acetone, 1.2 wt% butanol and 0.2 wt% ethanol	Total flux: 630.2	n-butanol SF: 17.4 PSI = 10,330 g/(m^2^ h)	[26]
1074	-	-	Phenol from water	80	1.5 wt% phenol	1500	78	[73]
2533	-	-	Phenol from water	60	1.5 wt% phenol	2016	40	[73]
2533	-	-	Phenol from water	70	1.5 wt% phenol	2133	64	[73]
2533 on a PVDF support	ZIF-L anisotropic nanosheets (ZLNs) (ZLN-0.05)	4	Phenol from water	50	1000 ppm phenol	Total flux: 4792; phenol permeance: 4.96 × 10^5^ GPU	14.4 phenol/water selectivity: 32.5	[37]
2533 on a PVDF support	-	-	Phenol from water	70	1000 ppm phenol	1.17	27.4	[39]
2533 on a PVDF support	Co-UMOFNs	40	Phenol from water	70	1000 ppm phenol	420	45.5	[39]
		40	Phenol from water	80	1000 ppm phenol	603	48.0	[39]
1657 on a PSf blend support	Graphene Oxide (GO)	0.4 for PSf	IPA dehydration	30	20 wt% water	1190	0 wt% IPA in permeate	[36]

**Table 6:** Pervaporation performance of Pebax-based MMMs for water purification (removal of organic molecules).

Pebax Grade	Filler Type	Loading (wt%)	Organic Separated from Water	T (°C)	Organic Conc. in the Feed	Permeation Flux	Separation Factor (SF)	Ref.
2533	MIL-53(Al)	15	Furfural	80	1 wt%	3800 g/(m^2^ h)	50.2	[38]
2533	Cu_2_O	6	Pyridine	30	1 wt%	89.3 g/(m^2^ h) (−13.1%)	10.2 (+22.8%)	[31]
2533	Cu_2_O	6	Pyridine	70	1 wt%	230 g/(m^2^ h)	18 PSI = 3910 g/(m^2^ h)	[31]
2533	MoS_2_	10	Pyridine	30	1 wt%	83.4 g/(m^2^ h) (−21.5%)	11.1 (+37.6%) PSI = 855 g/(m^2^ h) (+16%)	[33]
2533	MoS_2_	10	Pyridine	70	1 wt%	215 g/(m^2^ h)	17.1 PSI = 3462 g/(m^2^ h)	[33]
2533	NaX	2	Toluene	–	0.01 mg/L	80 g/(m^2^ h)	150 PSI = 11,500 g/(m^2^ h) Enrichment factor (β) = 920	[20]
2533	ZSM-5 (3- micron)	10	Ethyl acetate	50	5 wt%	200 g/(m^2^ h)	186	[22]
2533 on a PES support	ZSM-5 (average size of 35 nm)	10	Ethyl acetate	50	5 wt%	*laminar flow*: 1882 g/(m^2^ h)	125	[23]
2533 on a PES support	ZSM-5 (average size of 35 nm)	10	Ethyl acetate	50	5 wt%	*turbulent flow*: 1985 g/(m^2^ h)	134	[23]
2533 on a PVDf support	ILTf_2_N@MIL-101	7.5	Ethyl acetate	30	5 wt%	2853 g/(m^2^ h)	108 PSI = 304,900 g/(m^2^ h)	[28]
2533 on a PVDf support	ILTf_2_N@MIL-101	5	Ethyl acetate	30	5 wt%	2354 g/(m^2^ h)	208 PSI = 486,220 g/(m^2^ h)	[28]
2533 on a PSf-PES support	TiO_2_ nanoparticles	0.03 wt% TiO_2_ in the coating solution	Humic acid	-	20 ppm	75.32 L/(m^2^ h)	HA rejection: 98.22%	[28]

**Table 7:** Pervaporation performance of Pebax-based MMMs for gasoline desulfurization (thiophene removal from *n*-octane).

Filler Type	Loading (wt%)	T (°C)	Feed Conc. (ppm Thiophene)	Permeation Flux (kg/(m^2^ h))	Thiophene Enrichment Factor (β) (–)	Ref.
Hollow monocrystalline silicalite-1 (HMS) 200 nm	20	60	500	20.63 (+82% than Pebax membrane)	6.11 (+23% than Pebax membrane); PSI: 123 kg/(m^2^ h)	[21]
MoS_2_ 2D nanosheets	4	60	1312	11.42 (+22.3%)	9.11 (+65.9%)	[32]
Ag^+^@SNW	9	60	1312	16.35 (+78.5%)	6.8 (+30.0%)	[41]
PDA/GNS	6	40	1300	3.94 (+24%)	7.73 (+10%)	[35]
Ag-PDA/GNS	6	40	1300	4.42 (+40%)	8.76 (+25%)	[35]
Ag-PDA/GNS	6	70	1300	22.53	6.07	[35]
Hierarchical porous SBA-15	6	60	1312	22.07 (+30%)	6.76 (+50%)	[78]

All the membranes are thin-film composites and comprise a Pebax^®^2533-based MMM layer deposited on a PSf support.

**Table 8:** Performance of Pebax-based MMMs as dehumidifiers for PEMFCs.

Membrane	Filler Amount (wt%)	Vapour Permeability Coefficient, Pv (×10^4^ Barrer)	Air Permeability Coefficient, Pg (Barrer)	Vapour/Air Selectivity (×10^4^)	Ref.
Nafion 211	-	8.21	9.05	0.93	[48]
Nafion 212	-	16.21	5.89	2.80	[48]
Pebax^®^3533/PAA + CNC (fibre membrane)	4.8 (CNCs)	1.72	0.78	2.22 (+14%)	[48]
Pebax^®^1074 + MMT	2.9	7.7	0.47	16.4	[46]
Pebax^®^1074 + APOP-MMT	4.8	8.4	0.49	17.3	[46]
Pebax^®^1074 + IL@F-Ce	4	45.3	2.40 (100%)	18.9 (+83%)	[47]

1 Barrer = 1 × 10^−10^ cm^3^(STP) cm/(cm^2^ s cmHg), measure unit for permeability.

**Table 9:** Performance of Pebax-based MMMs for water treatment.

Pebax Grade	Filler Type	Loading (wt%)	Separation	Feed	Permeation Flux (L/(m^2^ h))	Rejection (%)	Ref.
1657 on a PES support	Multiwalled carbon nanotubes wrapped with chitosan (CWNTs)	1	Dye removal (Malachite green) from water	30 mg/L	13.9	96.7	[42]
		0.1		30 mg/L	6	98.7	[42]
2533 on a PSf support	Functionalized multiwall carbon nanotubes (F-MWCNTs)	0.5	Separation of oil/water emulsion by nanofiltration (TMP = 15 bar)	Oil/surfactant (3/1 wt/wt) in water	230	98.6	[43]
		2		Oil/surfactant (3/1 wt/wt) in water	174	99.3	[43]
1657	TiO_2_ nanotubes	10	Desalination under UV	Seawater	8.2 (+100%)	99.97 (salt)	[45]
